# Biopsy RNA-seq captures TROP-2–linked migration and clonal resistance to forecast aggressiveness in metastatic melanoma

**DOI:** 10.1186/s13046-026-03646-1

**Published:** 2026-01-28

**Authors:** Martina Betti, Celeste Accetta, Brindusa Ana Maria Arteni, Anya Rosselli, Elisa Melucci, Paolo Visca, Claudio Botti, Fabio Pelle, Michelangelo Russillo, Virginia Ferraresi, Emilia Migliano, Marianna Cerro, Stefano Scalera, Francesca De Nicola, Silvia Matteoni, Daniela Covino, Antonino Guerrisi, Matteo Pallocca, Maurizio Fanciulli, Edoardo Pescarmona, Giovanni Blandino, Rita Mancini, Italia Falcone, Simona Di Martino

**Affiliations:** 1 Biostatistics, Bioinformatics and Clinical Trial Center, IRCCS-Regina Elena National Cancer Institute, Rome, 00144 Italy; 2 Department of Computer, Control and Management Engineering (DIAG), Sapienza University of Rome, Rome, Italy; 3 Department of Pathology Unit, IRCSS-Regina Elena National Cancer Institute, Rome, 00144 Italy; 4 Department of Experimental Medicine, Sapienza University of Rome, Rome, Italy; 5 Department of Surgery, IRCCS-Regina Elena National Cancer Institute, Rome, 00144 Italy; 6 Sarcoma and Rare Tumors Departmental Unit, IRCCS-Regina Elena National Cancer Institute, Rome, 00144 Italy; 7 UOSD Plastic and Regenerative Surgery, San Gallicano Dermatological Institute IRCCS, Rome, 00144 Italy; 8 Department of Clinical and Molecular Medicine, Sapienza University of Rome, Rome, 00185 Italy; 9 Gene Expression and Cancer Models Unit, IRCCS-Regina Elena National Cancer Institute, Rome, 00144 Italy; 10 Radiology and Diagnostic Imaging Unit, Department of Clinical and Dermatological Research, San Gallicano Dermatological Institute IRCCS, Rome, 00144 Italy; 11 Istituto degli Endotipi in Oncologia, Metabolismo e Immunologia National Research Council, Naples, 80131 Italy; 12 Translational Oncology Research Unit, IRCCS-Regina Elena National Cancer Institute, Rome, 00144 Italy; 13 Department of Clinical and Molecular Medicine, Sapienza University of Rome, Rome, 00161 Italy

**Keywords:** Metastatic melanoma, Dormancy, TROP2, RNA-seq, Cell lines, Biomarkers, Immunotherapy

## Abstract

**Background:**

Unlike many other solid tumors, melanoma cells possess a remarkable ability to dynamically transition between distinct transcriptional states in response to environmental cues or therapeutic pressure. Among the adaptive mechanisms underlying this plasticity, the epithelial-to-mesenchymal transition (EMT) has garnered increasing attention, as it facilitates the shift from a proliferative, epithelial-like phenotype to a more invasive, mesenchymal-like phenotype frequently associated with cellular dormancy, quiescence and resistance to therapy. Despite growing interest in this phenomenon, the characterization of dormant cellular phenotypes and their clinical significance remains incomplete.

**Methods:**

In this study, we adopted a comprehensive approach integrating patient-derived melanoma cell lines, bulk RNA sequencing from tumor biopsies and analysis of independent bulk and single-cell public datasets. This multi-dimensional strategy enabled the identification of a reproducible dichotomy between “proliferative” and “dormant” phenotypes, characterized by distinct levels of mitotic activity and mesenchymal gene expression profiles. By leveraging an eight-gene transcriptional signature, we constructed a “dormancy score” able to stratify tumors along a dormancy–proliferation axis, revealing strong associations with clinical outcomes such as progression-free survival (PFS), overall survival (OS) and response to immunotherapy.

**Results:**

Within the dormant-associated gene module, the surface glycoprotein TACSTD2 (TROP2) emerged as a central hub gene. TROP2 expression was consistently upregulated in the dormant-like transcriptional state. Supporting these findings, single-cell RNA sequencing data confirmed that TROP2 marks a rare subpopulation of malignant cells that may constitute a quiescent, therapy-resistant niche. Besides, results highlight a predominant intracellular expression of TROP2 in the dormant phenotype.

**Conclusions:**

Together, these findings define a robust dormant phenotype in melanoma with both molecular and clinical significance and evaluate TROP2 as a potential biomarker and therapeutic target for identifying and eradicating dormant and treatment-refractory tumor cells.

**Supplementary Information:**

The online version contains supplementary material available at 10.1186/s13046-026-03646-1.

## Background

 Despite accounting for only 4–5% of all cutaneous malignancies [1], melanoma is responsible for most skin cancer-related deaths due to its aggressive biological behaviour and high metastatic potential [2, 3]. In recent years, the advent of targeted therapies such as BRAF and MEK inhibitors and immunotherapy has improved melanoma patient’s outcomes [4–6]. However, a considerable number of patients suffers disease relapse due to acquired or intrinsic resistance mechanisms [7, 8], emphasizing the need for a deeper understanding of the tumor’s adaptive landscape. Melanoma is a heterogeneous disease characterized by considerable transcriptional plasticity, which contributes to its growth, metastatic dissemination and treatment resistance [9–13]. In response to environmental pressures, such as therapeutic interventions, melanoma cells can dynamically transition between distinct transcriptional states and this capacity is primarily driven by non-genetic mechanisms such as chromatin remodelling, metabolic rewiring and lineage-specific transcriptional reprogramming [7, 14]. These transitions enable cells to adopt alternative phenotypic states without the need for new mutations, making them especially difficult to target therapeutically. The epithelial-to-mesenchymal transition (EMT) has emerged as a key process in enabling dynamic phenotypic switching in cancer cells [15]. In melanoma and other tumor contexts, EMT promotes the transition from a proliferative and epithelial-like phenotype to an invasive and mesenchymal-like phenotype. This process is frequently associated with reduced proliferative capacity, acquisition of drug resistance and cellular quiescence [15, 16], contributing to therapeutic failure and disease recurrence. This phenotypic plasticity underlies intra-tumoral heterogeneity and represents one of the major barriers to durable clinical responses [17, 18]. Different studies have delineated the role of specific transcriptional programs that modulating melanoma plasticity, revealing dynamic gene expression profiles that induce the transition between proliferative and invasive cell states [14, 19]. A complex network of transcription factors (such as MITF, SOX10, JUN), chromatin remodelling and microenvironmental factors regulate these processes. Despite these advances, a comprehensive understanding of dormant tumor cell phenotypes remains elusive. Dormant cells are characterized by reversible cell cycle arrest, survival capacity under stress and potential for later reactivation [20]. They can be key actors in minimal residual disease and late relapse. While certain transcriptional signatures have been proposed to mark dormancy-like states, a clearly defined and clinically actionable dormant state has yet to be fully established. Dormant melanoma cells may evade therapeutic surveillance and, therefore, they require novel treatment strategies aimed at reactivation or eradication through metabolic or epigenetic vulnerabilities. At this purpose, it is important better characterize dormancy-associated transcriptional and epigenetic landscapes, identify specific biomarkers and assess their prognostic value in clinical cohorts.

In this study, we used an integrative approach combining patient-derived melanoma cell lines, bulk RNA sequencing from tumor biopsies, and analysis of independent public datasets. This comprehensive strategy identified a clear biologically significant separation between “proliferative” and “quiescent” phenotypes, characterized by distinct levels of mitotic activity and mesenchymal gene expression. We developed a “dormancy score” that stratifies tumors along a continuum from dormancy to proliferation. This score demonstrated strong correlations with key clinical endpoints, including progression-free survival (PFS), overall survival (OS) and response to immune checkpoint blockade, as validated in the Dana-Farber Cancer Institute (DFCI) 2019 cohort. Trophoblast cell surface antigen 2 (TACSTD2 or TROP2) emerged as a key gene within the dormancy program, consistently elevated in dormant-like tumors with concordant RNA and protein expression across datasets. Single-cell data showed that TROP2 marks a rare, quiescent, therapy-resistant malignant subpopulation. Protein analyses further revealed predominantly intracellular TROP2 localization in dormant melanomas, adding an additional layer of stratification. Collectively, our findings define a transcriptionally distinct and clinically relevant dormant state in melanoma and highlight TROP2 as a promising biomarker and therapeutic target for the identification and elimination of dormant, treatment-refractory tumor cells.

## Methods

### Cohort

A cohort of 26 melanoma patients was selected for integrative transcriptomic analysis, including 7 individuals harboring NRAS mutations,18 with BRAF mutations and one wt case, as detailed in Table 1.

**Table 1:** Cohort description

ID	Age at Diagnosis	Gender	Driver Mutation	Surgery Location	Diagnosis	Stage	T
BBIRE-T 1652	75	F	NRAS p.Q61R	In-transit Metastasis	Melanoma	IIIB	4b
BBIRE-T 1773	74	F	NRAS p.G13D	In-transit Metastasis	Melanoma	IIB	4b
BBIRE-T 1841	55	F	BRAF p.V600E	In-transit Metastasis	Melanoma	IIIC	3b
BBIRE-T 1848	36	F	BRAF p.V600E	In-transit Metastasis	Melanoma	IIIC	3b
BBIRE-T 1918	48	F	BRAF p.V600E	In-transit Metastasis	Melanoma	IA	1a
BBIRE-T 1957	72	F	BRAF p.V600E	In-transit Metastasis	Melanoma	IV	4b(m)
BBIRE-T 1978	39	M	BRAF p.V600E	In-transit Metastasis	Melanoma	IB	2a
BBIRE-T 1995	54	F	BRAF p.V600K	In-transit Metastasis	Melanoma	IIIC	4b
BBIRE-T 2061	60	M	NRAS p.Q61K	In-transit Metastasis	Melanoma	IB	2a
BBIRE-T 2074	61	M	NRAS p.Q61K	In-transit Metastasis	Melanoma	IIC	4b
BBIRE-T 2108	66	F	BRAF p.V600E	In-transit Metastasis	Melanoma	IIIC	3b
BBIRE-T 2177	55	M	BRAF p.V600E	In-transit Metastasis	Melanoma	IIIA	2a
BBIRE-T 2217	59	M	BRAF p.V600E	In-transit Metastasis	Melanoma	IV	x
BBIRE-T 2263	55	M	BRAF p.V600K	In-transit Metastasis	Melanoma	IIB	3b
BBIRE-T 2371	76	F	NRAS p.Q61L	In-transit Metastasis	Melanoma	IIIC	3b
BBIRE-T 2393	55	M	BRAF p.L597S	In-transit Metastasis	Melanoma	IIIB	2b
BBIRE-T 2450	63	M	BRAF p.V600K	In-transit Metastasis	Melanoma	IIID	4b
BBIRE-T 2482	55	M	BRAF p.V600E	In-transit Metastasis	Melanoma	IV	x
BBIRE-T 2523	78	M	NRAS p.Q61K	In-transit Metastasis	Melanoma	IIIB	3a
BBIRE-T 2542	59	M	BRAF p.V600E	In-transit Metastasis	Melanoma	IV	4b
BBIRE-T 2546	56	M	BRAF p.V600E	In-transit Metastasis	Melanoma	IV	3b
BBIRE-T 2569	85	F	NRAS p.Q61L	In-transit Metastasis	Melanoma	IIIC	4a
BBIRE-T 2615	73	F	BRAV p.V600E	In-transit Metastasis	Melanoma	IIID	4b
BBIRE-T 2648	73	M	BRAF p.V600E	In-transit Metastasis	Melanoma	IIC	T4b
BBIRE-T 2658	83	F	WT	In-transit Metastasis	Melanoma	IV	t4a
BBIRE-T 2691	86	F	BRAF p.D594N	In-transit Metastasis	Melanoma	NA	t3b

Basic clinical description of patients

**Fig. 1:**
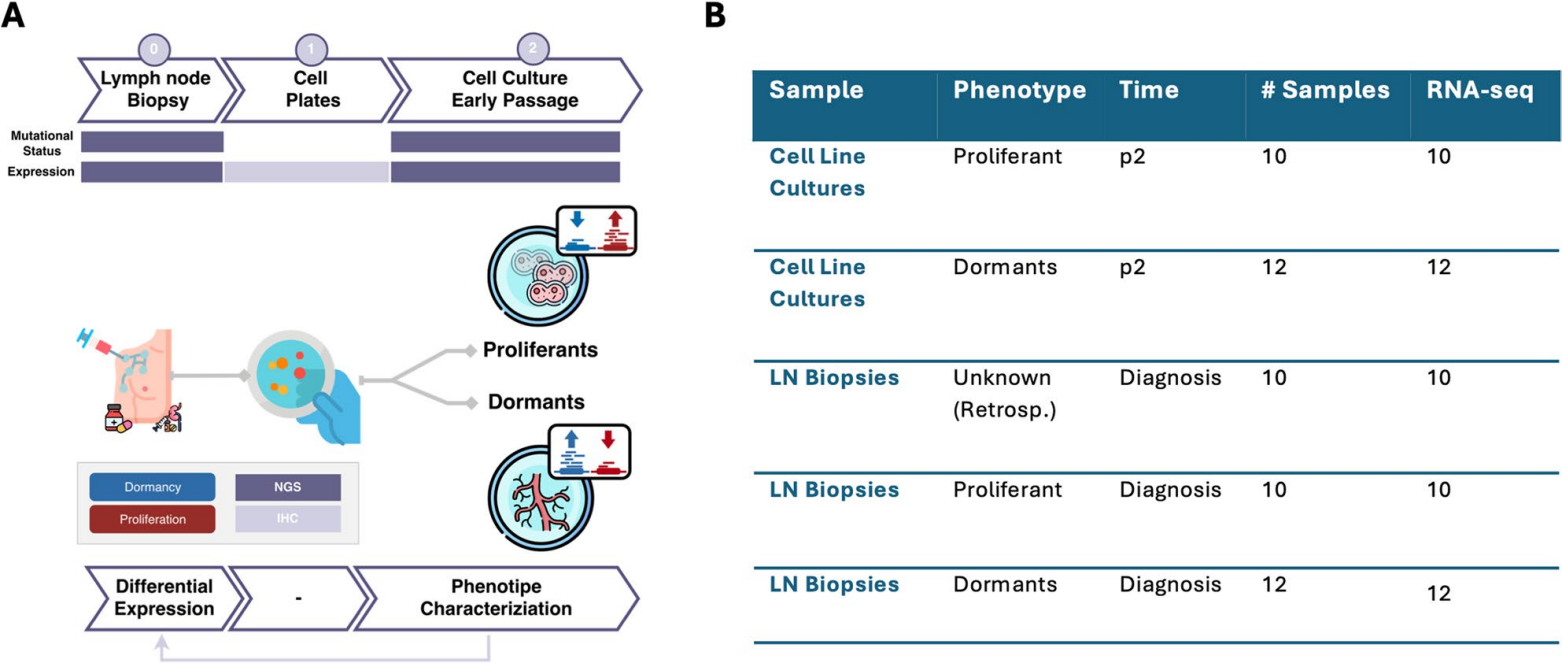
Study Design. **A** Sample Collection Design. Dark purple represents the steps at which NGS analysis was performed, either at DNA or RNA level; light purple represents the steps at which either IHC or PCR were performed. **B** Overview of tissue derived samples and corresponding cell lines

At the time of lymph node biopsy, all patients underwent comprehensive molecular profiling through targeted next-generation sequencing (NGS) and RNA sequencing (RNA-seq). For a subset of these samples, primary tumor cell cultures were successfully established (*n* = 22). Target NGS analysis was performed on the derived cell lines to confirm genomic concordance with the corresponding tumor tissues, thereby ensuring the fidelity of the in vitro models. RNA-seq was subsequently conducted on early-passage (passage 2) cell lines to capture transcriptional states reflective of the original tumor. All these steps are summarized in Fig. 1A. Additionally, an independent group of 10 BRAF-mutant patients was included in the study; although matched cell lines were not established for these cases, RNA-seq was performed directly on their lymph node biopsies. A summary of successfully profiled samples and experimental conditions is shown in Fig. 1B. Finally, a comparative overview of mutational profiles between patient tumors and matched cell lines is shown in Table 2.

**Table 2:** Samples mutational status

Sample ID	Biopsy (Diagnosis)				Cell Line (p2)
	Mutation (DNA)	VAF%	Mutation (RNA)	VAF%	Mutation (DNA)
BBIRE-T 2691	BRAF D594N	37	BRAF D594N	61	BRAF D594N
BBIRE-T 2393	BRAF L597S	61	BRAF L597S	65	na
BBIRE-T 1957	BRAF V600E	83	BRAF V600E	31	WT
BBIRE-T 2217	BRAF V600E	na	BRAF V600E	72	BRAFV600E
BBIRE-T 1978	BRAF V600E	20	BRAF V600E	68	BRAF V600E
BBIRE-T 2546	BRAF V600E	34	BRAF V600E	38	BRAF V600E
BBIRE-T 1848	BRAF V600E	28	BRAF V600E	30	WT
BBIRE-T 1918	BRAF V600E	62	BRAF V600E	63	WT
BBIRE-T 2482	BRAF V600E	60	BRAF V600E	75	WT
BBIRE-T 1841	BRAF V600E	53	BRAF V600E	43	WT
BBIRE-T 2615	BRAF V600E	32	WT	-	BRAF V600E
BBIRE-T 2648	BRAF V600E	47	BRAF V600E	70	BRAF V600E
BBIRE-T 1995	BRAF V600K	72	BRAF V600K	87	BRAF V600K
BBIRE-T 2450	BRAF V600K	34	BRAF V600K	55	na
BBIRE-T 2108	BRAF V600E	37	BRAF V600E	50	BRAF V600E
BBIRE-T 2177	BRAF V600E	25	BRAF V600E	46	WT
BBIRE-T 2263	BRAF V600K	na	BRAF V600K	67	WT
BBIRE-T 1773	NRAS G13D	16	NRAS G13D	80	WT
BBIRE-T 2061	NRAS Q61K	42	NRAS Q61K	59	NRAS Q61K
BBIRE-T 2074	NRAS Q61K	29	WT	-	WT
BBIRE-T 2523	NRAS Q61K	27	NRAS G13D	44	WT
BBIRE-T 2569	NRAS Q61L	43	NRAS G13D	42	na
BBIRE-T 2371	NRAS Q61L	na	NRAS Q61L	54	NRAS Q61L
BBIRE-T 1652	NRAS Q61R	27	NRAS Q61R	33	NRAS Q61R
BBIRE-T 2542	BRAF V600E	na	WT	-	na
BBIRE-T 2658	WT	-	-	-	WT

Variants found on the DNA and RNA of the same samples with their associated frequency of mutation if applicable. Mutations found on corresponding cell lines when applicable

### Cell cultures

Primary human melanoma cell cultures were established and characterized by the Regina Elena National Cancer Institute Biobank (BBIRE-T). All cell lines were derived from excess tumor tissue obtained during lymphadenectomy procedures for surgically resected melanoma metastases, following written informed consent from the patients. Tumor samples were subjected to both mechanical (scissors/scalpels) and enzymatic (Collagenase Type II) dissociation to obtain single-cell suspensions. The resulting cell preparations were cultured in RPMI-1640 medium supplemented with 10% heat-inactivated fetal bovine serum (FBS), L-glutamine, and penicillin-streptomycin, and maintained in a humidified incubator at 37 °C with 5% CO₂. All established cell lines were routinely tested for mycoplasma contamination using a PCR-based assay. Clinical diagnosis and molecular characterization of the tumor material were confirmed through histopathological evaluation, immunohistochemistry, and target sequencing (NGS) analysis.

### RNA extraction and preservation

Total RNA from Formalin-fixed, paraffin-embedded (FFPE) tissue sections were extracted using QIAGEN RNeasy FFPE Kits (Qiagen, IT). FFPE blocks were cut into 4–5 -µm slices and placed in conical-tube for the deparaffinization with QIAGEN Deparaffinization solution, designed for removing paraffin from FFPE tissue sections before DNA or RNA extraction, allowing for more reliable downstream applications. Total RNA (including miRNAs) from cell pellets were extracted using miRNAeasy mini-Kits (Qiagen, IT). QIAzol lysis Reagent was added to the samples for disrupt and homogenate cells, according to the manufacturer’s instructions. Quantity and integrity of the extracted RNA were assessed by NanoDrop Spectrophotometer (NanoDrop Technologies, DE) and by Agilent TapeStation (Agilent Technologies, CA), respectively.

### Bulk RNA-seq

RNA libraries for sequencing will be generated using the same amount of RNA for each sample according to the Illumina Stranded Total RNA Prep kit with an initial ribosomal depletion step using Ribo-Zero Plus (Illumina, CA). The libraries will be quantified by qPCR and sequenced in paired-end mode (2 × 100 bp) with NovaSeq 6000 (Illumina, CA). For each sample generated by the Illumina platform, a pre-process step for quality control will be performed to assess sequence data quality and to discard low-quality reads.

### DNA extraction and target next-generation sequencing (NGS)

Genomic DNA was extracted using the QIAamp DNA FFPE Tissue Kit on the QIAcube^®^ automated platform (Qiagen), following the manufacturer’s instructions. DNA quantification was performed using a Qubit Fluorometer (ThermoFisher Scientific) with the Qubit^®^ dsDNA HS Assay Kit (ThermoFischer Scientific). Library preparation was carried out using 10 ng of input DNA (range 1–20 ng) with the Ion AmpliSeq Library Kit 2.0 (ThermoFisher Scientific). Targeted sequencing was performed using the Ion AmpliSeq™ Cancer Hotspot Panel v2 (CHPv2) (ThermoFisher Scientific), which includes 207 amplicons covering ~ 2,800 known mutations from the COSMIC database across 50 oncogenes and tumor suppressor genes. Library barcoding and dilution were automated using the Ion Chef Instrument, along with the Ion Code Barcode 1–32 Kit (Thermo Fisher Scientific). Libraries were normalized to 100 pM, pooled in equimolar concentrations and diluted to 35 pM for template preparation. Template preparation was performed by the Ion Chef system (Termofisher Scientific), which integrates library amplification, Ion Sphere Particles (ISP) recovery-enrichment and Chip loading. Sequencing was performed on Ion S5 system (Termofisher Scientific), with the Ion 530 chips. Analysis was carried out using Ion Torrent Suite™ Software version 5.18.1 and Ion Reporter™ version 5.20.2.0. The Torrent Suite™ Software was used to perform initial quality control including chip loading density, median read length and number of mapped reads. The Coverage Analysis plugin was applied to all data and used to assess amplicon coverage for regions of interest. Variants were identified by Ion Reporter filter with a detection threshold of 5% variants. A cut-off of 500X coverage was applied to all analyses. Only single nucleotide variants (SNVs) resulting in a nonsynonymous amino acid change, or a premature stop codon, and all short indels resulting in either a frameshift or insertion/deletion of amino acids were selected. All variants were manually reviewed with Integrative Genomics Viewer (IGV v.2.8.0, Broad Institute, Cambridge, Massachusetts, USA) and with the support of publicly available datasets reporting on their established or predicted oncogenicity (i.e., COSMIC, cBioPortal, Clinical Trials, ClinVar, dbSNP, dbVar, Catalog of somatic mutations in cancer, My Cancer genome, personalized cancer therapy, NCBI genome, RefSeqGene, and Locus reference Genomic). For further examination of NGS data, to prioritize variants and find the relevant cancer drivers, we used the genomic analysis software “Oncomine Reporter”.

### Digital pathology

FFPE sections were immunostained for the two candidate biomarkers TROP2 (TACSTD2/TROP2 (F4W4J) Rabbit mAb #76730- Cell Signaling Technology) and SOX9 (SOX9 (D8G8H) Rabbit mAb #82630-Cell Signaling Technology). Whole-slide images were then captured on the Aperio GT 450 DX scanner at 40× magnification, yielding 0.26 μm/pixel virtual slides whose colour-calibrated optics preserve sub-micron histologic detail (Leica Biosystems). Each virtual slide was evaluated by a pathologist, delineating viable tumor regions and scoring staining intensity and cellular localization to distinguish malignant from benign elements. Cells were annotated as positive only when they showed unequivocal membranous or nuclear labelling for the predefined immunohistochemical markers.

### Immunoflorescence staining and confocal microscopy

Thirty thousand proliferative and dormant cells were plated, and after 48 h the cells were fixed with 4% paraformaldehyde for 10 min, followed by permeabilization in PBS containing 0.1% Triton X-100 for 10 min. After blocking in 5% bovine serum in PBS for 1 h, cells were incubated with an anti-Ki-67 monoclonal antibody (D3B5, #9129; Cell Signaling Technology, Danvers, MA, USA) diluted 1:400, for 90 min at room temperature (RT). Subsequently, samples were incubated with Alexa Fluor 488–conjugated goat anti-rabbit secondary antibody (A11034; Thermo Fisher Scientific) diluted 1:500, together with Alexa Fluor 555 Phalloidin (8953; Cell Signaling Technology), both at RT. Nuclei were counterstained with DAPI before imaging. Immunofluorescence analyses were performed using an LSM 880 confocal laser scanning microscope equipped with an AiryScan detector (Carl Zeiss AG, Oberkochen, Germany) and a 40× oil-immersion objective. Samples were excited using 405 nm and 488 nm laser lines. Fluorescence images were acquired from multiple fields per sample using Zen software (Carl Zeiss, Germany). For Ki67 staining, the software automatically generated regions of interest (ROIs) around individual nuclei, and the mean Ki67 fluorescence intensity within each nucleus was subsequently exported. For phalloidin staining, mean fluorescence intensity was quantified using Zen 3.12 (Zen Desk) and exported to GraphPad Prism (GraphPad Software) for statistical analysis and data visualization.

### FACS analysis

Tissues were dissociated to single-cell suspensions by combining mechanical and enzymatic dissociation as previously described. Cells obtained were washed and stained with LIVE/DEAD fixable Violet Dead Cell (Cat. L34963 Thermo Fisher) stain to assess viability. To label TROP2 expressed in surface and immune fraction, cells were incubated for 20 min at 4 °C with TROP2 (Cat. ABS380, clone (01), Enzo) or CD45/BV510 human antibody (Cat. 563204, clone BHI30, BD Biosciences). For detection of intracellular TROP2 and S100, cells were fixed with 4% formaldehyde (Cat. 415661, Carlo Erba) for 10 min, permeabilized with 0.2% Triton X-100 (Cat. 9002-93-1 Biotech) in PBS with 1% BSA (Cat. 9048-46-8, Thermo Fisher) 10 min at room temperature, incubated for 30 min with anti-TROP2 (Cat. A214488, clone EPR20043, abcam) and anti-S100 antibody (Leica, S100-167-L-CE) and then, with secondary antibody, goat anti-mouse Alexa Fluor 647 (Cat. A-21235) or (Cat. A1101) goat anti-rabbit Alexa Fluor 488 IgG (Thermo Fisher Scientific) respectively for 30 min at room temperature. Positive cell gating was set using fluorescence minus one control (FMO) or the only secondary antibody. Data analyses were performed using FlowJo v10 (BD Biosciences).

### Western blot analysis

To obtain whole protein extract, cells were lysed in SDS lysis buffer containing 20 mmol/L Tris-HCl (pH 8.0), 100 mmol/L NaF, 1 mmol/L NaVO_4_, 10 mmol/L PMSF, 10 µg/mL leupeptin, 2% SDS. Protein concentration was detected by Bio-Rad Protein Assay Dye Reagent Concentrate (Bio-Rad, Hercules, CA, USA). Approximately 20 ng of total proteins were fractionated by SDS-polyacrylamide gel electrophoresis and transferred to the nitrocellulose membrane (Amersham, Arlington Heights, IL, USA). Membrane was probed with specific primary antibody for p-TROP2 (Cell Signaling Technology Inc., Beverly, MA, USA Cat#79865). To control the number of proteins transferred to the nitrocellulose membrane, GAPDH (Cell Signaling Technology Inc., Beverly, MA, USA Cat#5174) was used. Membrane was incubated with peroxidase-conjugated anti-rabbit secondary antibodies (Jackson Immunoresearch Labs, Inc., Baltimore, MD, USA) and developed with a chemiluminescence (ECL) system (Amersham). For image detection, UVITEC Alliance 4.7 system (Cambridge, UK) was used.

### Primary bioinformatics analysis

RNA-seq quantification was performed using the *nf-core/rnaseq* pipeline [21]. RNA variant calling was carried out using the *nf-core/sarek* and *nf-core/rnavar* pipeline [21]. Diagnostic variant calling for clinical samples employed proprietary Ion Torrent Suite Software (Thermo Fisher Scientific) in accordance with institutional protocols.

### Secondary analysis

Differential Expression Analysis was performed via *DESeq2* [22] to identify significantly differentially expressed genes between comparison groups. Standard PCA analysis was performed via *sklearn.decomposition*, while accuracy metrics were computed via *sklearn.metrics*. Co-expression Network Analysis was performed via *DWGCNA* to construct and analyse weighted gene co-expression networks, enabling detection of key regulatory modules. For functional Enrichment, *Enrichr* [23] was utilized for Gene Ontology and pathway enrichment. Finally, in-Silico Deconvolution for the estimation of Immune and stromal cell type proportions was run via *CibersortX* [24] using the standard LM22 single cell-derived cell markers [25] with 100 permutations and batch normalization. All the analyses were conducted via R, bash and Python scripting.

### Tertiary analysis

Transcript-level expression (TPM) was normalized and transformed into z-scores for gene-signature scoring. Kaplan–Meier rank statistics were derived for survival associations using publicly available datasets; log-rank tests were applied to evaluate differences in survival curves. Graph-Based Visualization and Clustering was performed using *igraph* [26] for facilitated network visualization and detection of highly connected modules. Signature classification performance on training sets was assessed using F1 score, precision, recall, and accuracy metrics. All the analyses were conducted via R and Python scripting.

### Validation on external bulk RNA-seq and single cell cohorts

For assessing the prognostic value and the numerical stability of our signature, we selected bulk RNA-seq dataset of primary tumor biopsies from the Metastatic Melanoma (DFCI, Nature Medicine 2019) via cBioPortal. To conduct further validation about the specificity of the identified biomarker, we included two external single cell datasets obtained from the Broad Institute Single Cell Portal (https://singlecell.broadinstitute.org/single_cell): (i) SCP109 dataset includes malignant cells from ~ 30 patients with advanced melanoma treated with immune checkpoint inhibitors [27] (ii) SCP1493 cohort comprises 32 fresh melanoma brain-metastasis samples from patients receiving immune checkpoint inhibitors [28]. At the protein level, expression patterns were verified using data from The Human Protein Atlas [29], allowing further correlation of RNA signatures with proteomic findings.

### Statistical analysis

Comprehensive statistical methods were applied, including standard descriptive statistics (mean, median, standard deviation), hypothesis testing (wilcoxon or nonparametric equivalents), and multivariate analyses (Cox proportional hazards models for survival). Principal Component Analysis (PCA) was performed to select genes that better captured numerical variability in the dataset. Bonferroni adjustment was applied to account for multiple comparisons in the differential expression and enrichment analyses. All the analyses were conducted via R and Python scripting and GraphPad Prism.

## Results

### Cell lines characterization and phenotype description

Primary melanoma cell lines were established from metastatic lymph nodes surgically resected from melanoma patients. Following in vitro culture for approximately 10 days, a group of these lines retained the phenotypic features and mutational landscape of the original tumor tissue, including the presence of the previously identified driver mutation. In contrast, other cell lines showed a loss of characteristic melanoma markers, and the driver mutation was no longer detectable by NGS. This apparent “loss” of mutations is most likely due to the low frequency of mutant cells within the cultured population, potentially falling below the analytical sensitivity threshold of the NGS platform employed, furthermore the wt cell lines presented a phenotype aligned with the lack of mutations that drive proliferation (Figure S1).

**Fig. 2:**
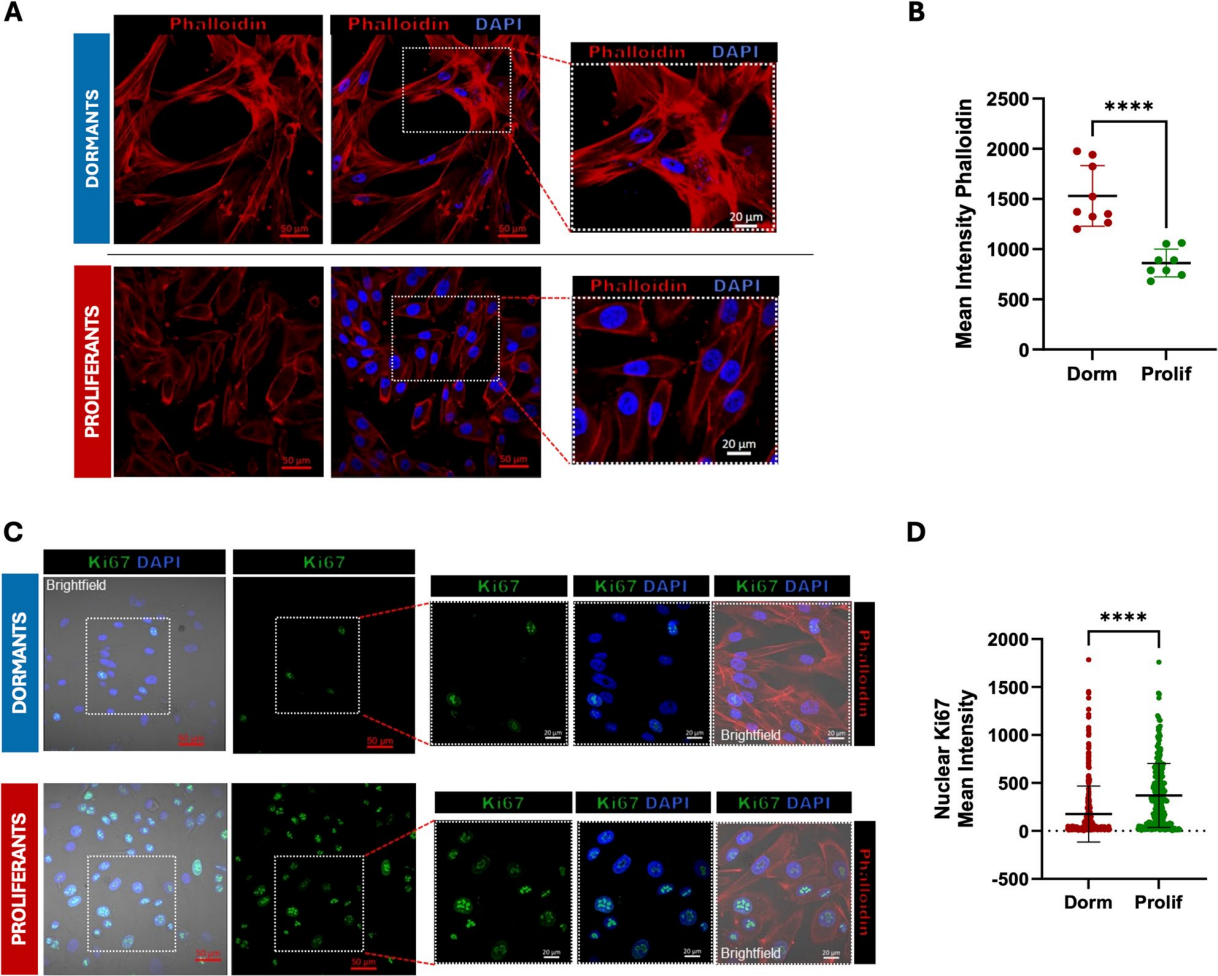
Phenotypic and Morphological Characterization of *dormant* and *proliferative* Phenotypes. **A**-**B** Phenotypic and morphological characterization of dormant and proliferent samples cells via immunofluorescence and corresponding Phalloidin quantification (**C**-**D**) Proliferation characterization of dormant and proliferent samples cells via immunofluorescence and corresponding nuclear Ki67 quantification

At our institution, the Department of Surgery has implemented a structured, multidisciplinary workflow that integrates the institutional biobank (BBIRE) as a central hub for both clinical management and translational research in metastatic melanoma. In close collaboration with the Department of Pathology, the surgical team ensures the standardized collection, processing and annotation of high-quality biological specimens. These biospecimens are critical for enabling advanced molecular studies, including NGS-based profiling, integrative bioinformatics and multi-omics analyses, thereby supporting precision oncology initiatives. Figure 1A illustrates the “Sample Collection Design”, a stepwise framework designed to optimize the acquisition, processing and utilization of tumor samples. Each workflow stage is meticulously planned to ensure maximal preservation of sample integrity and data quality. This comprehensive approach enables the generation of robust genomic and transcriptomic datasets, facilitating the identification of driver mutations, gene expression patterns and other molecular alterations critical for the development of personalized therapeutic strategies. All tissue specimens and their corresponding in vitro models are summarized in the in Fig. 1B. Primary melanoma cell cultures were successfully established and characterized. Microscopic evaluation revealed two distinct phenotypic states across the cell lines: a dormant, mesenchymal-like morphology consistent with undifferentiated and potentially invasive behaviour and a proliferative, melanocytic phenotype characterized by large flattened melanocyte-like cells. These observations were consistently replicated across multiple melanoma cultures. Confocal imaging, performed on two representative cell lines, confirmed the marked morphological and proliferative differences between the dormant mesenchymal-like and the proliferative melanocytic phenotype (Fig. 2A).Confocal imaging of filamentous actin (F-actin) stained with phalloidin revealed a distinct redistribution of filaments between the two different phenotypes: in dormant cells, filaments were considerably longer and formed an extensive network, a pattern absent in proliferant cells (Fig. 2A). Quantitative analysis further confirmed increased phalloidin intensity in dormant cells, supporting previous observations of enhanced actin fiber organization in resistant cells compared with melanoma controls as shown in Fig. 2B [30]. Another key feature distinguishing the proliferant and dormant phenotypes is their differing levels of mitotic activity. The nuclear marker Ki-67 is commonly used to assess proliferative status (Fig. 2C). Confocal analysis of Ki-67 revealed that the marker was detectable in both phenotypes. While a subset of dormant cells still displayed Ki-67 staining, the proportion of Ki-67–positive nuclei was markedly higher in proliferating cells, indicating that dormant cells exhibit a substantially reduced proliferative capacity compared with their proliferating counterparts (Fig. 2D). Moreover, the distinction between the dormant and proliferative phenotypes was further substantiated by Ki-67 immunostaining performed on cell-block, which consistently recapitulated the markedly lower proliferative activity characteristic of dormant cells (Figure S2A-B) compared with proliferating ones (Figure S2C-D).

Transcriptomic analysis revealed significant differences between the two phenotypes in vitro. Differential gene expression analysis identified the EMT pathway as the most prominently upregulated signature in the dormant mesenchymal-like cells (Figure S3). This finding suggests that EMT may play a key role in maintaining the invasive and therapy-resistant characteristics of non-proliferative melanoma cell populations, highlighting potential molecular targets for future therapeutic intervention.

### Oncogenic mutations confirmed at the transcription level

All tumor samples were initially characterized using targeted DNA sequencing and the identified mutations along with their respective variant allele frequencies (VAFs) are summarized in Table 2.

To assess whether the DNA-level mutations were transcriptionally active, we performed variant calling on RNA-seq data and compared the mutant allele fractions at the transcript level to the corresponding DNA VAFs. Among the 17 tumors harbouring BRAF mutations, the mutation was confirmed at the RNA level in all but three cases, presumably due to unfeasibility of transcript-level quantification. Among BRAF-mutated cases, DNA VAF was available for 14; in 12, the RNA VAF exceeded the DNA VAF, whereas in 2 it was lower. These findings are consistent with known copy number gains at the BRAF locus, which can result in elevated mutant transcript levels. Similarly, of the seven tumors with an NRAS DNA mutation, four showed the identical mutation at the RNA level (with higher RNA VAF), whereas two displayed an RNA variant at a different NRAS site (G13D), supporting reliable transcription of mutant alleles across both oncogenes.

### Transcriptomic analysis identifies a dormancy signature associated with poor prognosis

**Fig. 3:**
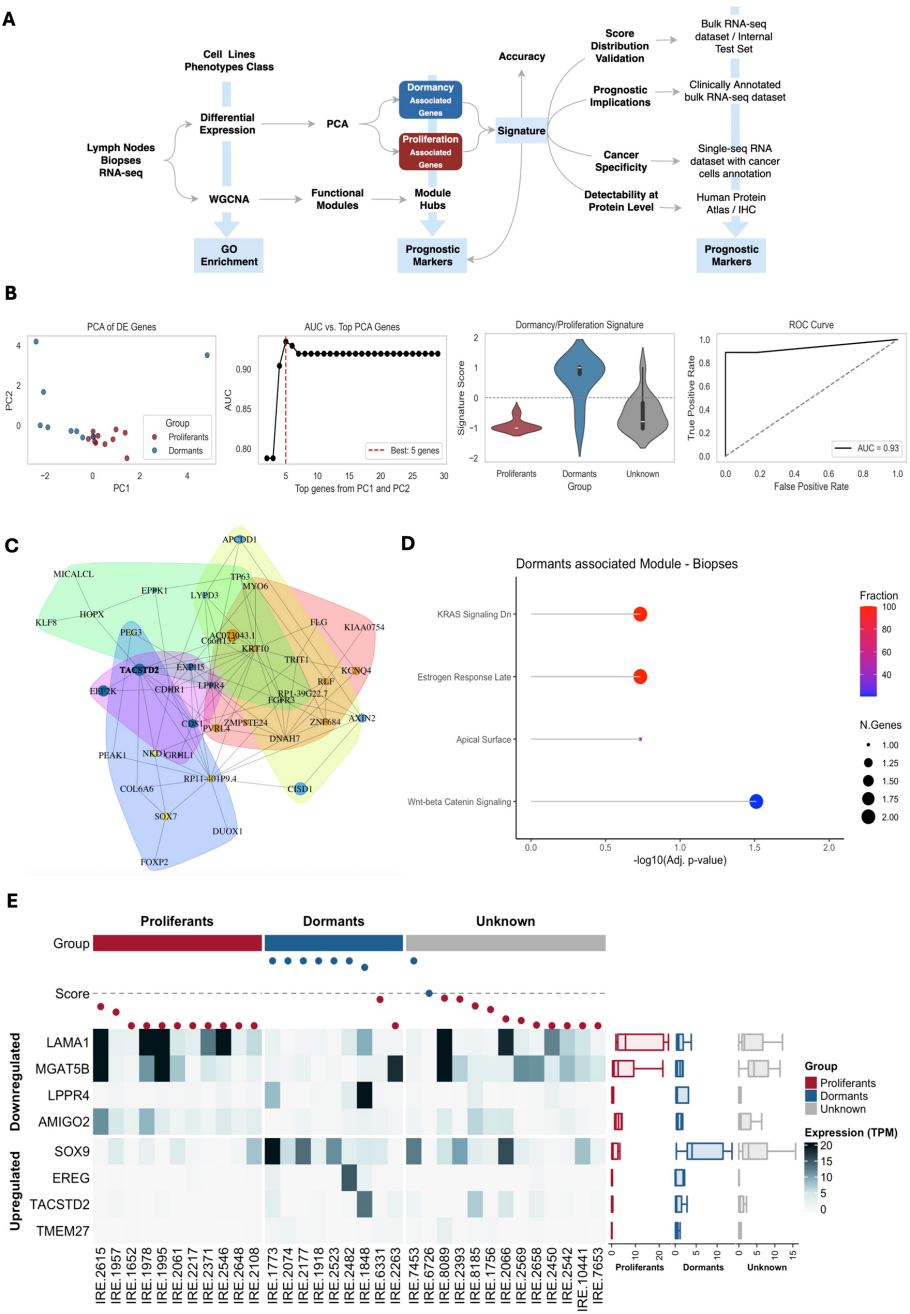
Analytical Workflow for Signature Identification. **A** Analytical design on the internal discovery cohort and validation on internal and external cohort. In light blue boxes are represented the main outcomes: phenotype description, transcriptomic signature and prognostic biomarkers. **B** PCA based on individual samples expression levels of DE genes; cutoff selection strategy for gene-signature computation; derived signature distributions per group; AUC obtained using a zero-threshold based classification into predicted dormants or proliferants (**C**) Gene module positively associated with the dormant phenotype in biopsies-derived samples. Nodes represent genes, nodes size represent central degree; nodes colour represents which cluster each node belongs to. **D** Functional enrichment of genes in the module represented in (**C**) (Hallmark DB 2021). **E** Signature heatmap. Individual gene expression level and signature scores from − 1 to 1 with color-coded classification (dot colour) and ground truth annotation (top bar); right boxplot annotation indicates gene-level expression variability per group

**Fig. 4:**
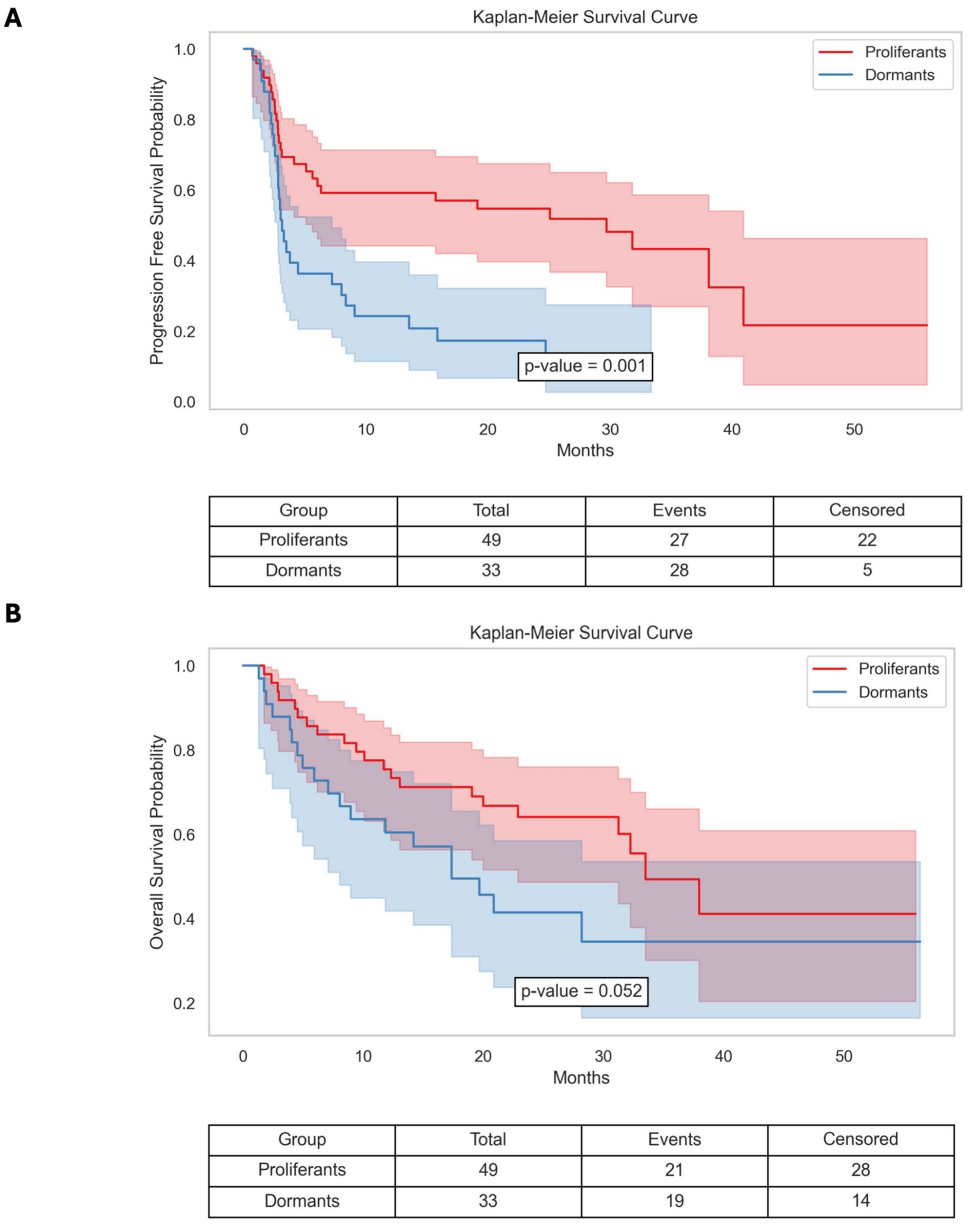
Prognostic Signature. **A** Progression Free Survival in DFCI 2019 cohort for patients stratified according to the proposed gene-expression signature (**B**) Overall Survival in DFCI 2019 cohort for patients stratified according to the proposed gene-expression signature

Differential expression analysis of RNA-seq data (Table S1) from paired proliferative and dormant melanoma cell line models (Fig. 3A) identified over 80 genes that were significantly regulated. Among these, 8 genes (TACSTD2, EREG, SOX9, LPPR4, LAMA1, TMEM27, AMIGO2 and MGAT5B) showed the highest discriminative potential across the two groups, as highlighted by the PCA analysis (Fig. 3B – PCA of DE genes and AUC). To quantify the dormancy phenotype at the transcriptomic level, Z-scoring each transcript and computing the normalized difference between the summed up-regulated (TACSTD2, EREG, SOX9 and LPPR4) and down-regulated components (LAMA1, TMEM27, AMIGO2 and MGAT5B) produced a composite dormancy score that spans − 1 (*proliferative*) to + 1 (*dormant*), with zero as the decision boundary (Fig. 3B – Signature Score). This signature scored 0.93 AUC score (Fig. 3B – ROC curve) and enables us to verify phenotypes observed in vitro. To further dissect the transcriptional dormancy phenotype background, we performed a weighted gene correlation network analysis (WGCNA) on the same RNA-seq dataset. This analysis identified a single eigengene module (Fig. 3C) positively correlated with dormancy scores (*r* = 0.6, *p* = 0.01). Gene Ontology enrichment revealed this module was significantly associated with pathways involved in negative regulation of cell proliferation, and positive regulation of cell- migration and differentiation (Fig. 3D). The hub gene of this dormancy-associated module was identified as TROP2, already observed in the first analysis, a known surface marker previously implicated in tumor aggressiveness and epithelial plasticity [31, 32]. The dormancy signature was applied to (i) an independent set of 18 previously unclassified melanoma biopsies (Fig. 3E, “Unknown”) and (ii) the publicly available *DFCI 2019* metastatic melanoma cohort (*n* = 121; Fig. 4A-B, with corresponding censoring tables). In both datasets, the distribution of dormancy scores yielded a certain proportion of dormant-like samples centered within the expected range (around 50%), confirming the generalizability of the scoring model.

Moreover, the *DFCI 2019* cohort, Kaplan–Meier survival analysis revealed that patients with a dormant-like transcriptional phenotype experienced significantly shorter progression-free intervals (*p* = 0.01) and a strong implication on OS (*p* = 0.052) compared to those classified as proliferant-like (Fig. 4A-B). These findings suggest that the dormancy program, while transcriptionally resembling a non-proliferative state, marks melanomas with more aggressive clinical behavior. In support of this, response rates to immunotherapy, as assessed by RECIST criteria, were significantly lower in the dormant group (χ² = 9.97, *p* = 0.007; Figure S4A).

Notably, no significant difference was observed in CD8 + T cell infiltration between dormant and proliferative phenotypes (Figure S4B), suggesting that immunotherapy resistance in the dormant state may be independent of cytotoxic immune cell exclusion. To validate these observations experimentally, we performed IHC on tissue sections from two dormant and two exemplary proliferative samples, assessing CD8 and CD20 expression to evaluate whether immune infiltration patterns could explain the transcriptional differences linked to TROP2 [33, 34] these patterns do not support a systematic depletion of cytotoxic T cells in *dormants* lesions (Figure S4C). CD20⁺ B-cell infiltrates were also detected, showing higher peritumoral abundance in proliferative samples. This pattern is consistent with reports linking CD20⁺ aggregates and tertiary lymphoid structure formation to more potent antitumor immune responses [35]. Taken together, these findings confirm that immune infiltration is present across both phenotypic groups and does not show a pattern suggestive of immune exclusion in *dormants* or *proliferants* samples. While we do not draw immunological conclusions beyond this descriptive assessment, our data support the notion that the *dormant* phenotype we identify is not redundant with, nor primarily driven by, major shifts in immune infiltration.

### Single-cell analysis shows a heterogeneous expression of dormancy-associated genes

**Fig. 5:**
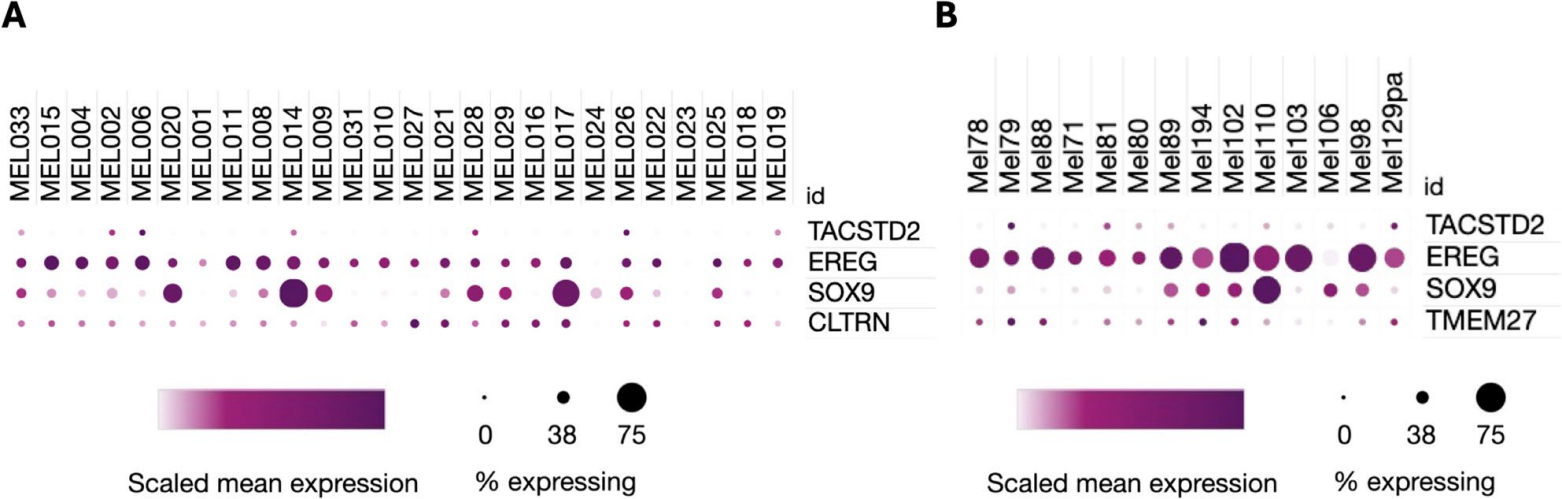
Single cell expression in dormancy-associated genes. **A** Dataset SCP109 expression of TACSTD2, EREG, SOX9 and TMEM27 (CLTRN). Color indicates normalized TPM expression; size indicates % of cells expressing a specific gene within a given sample. **B** Dataset SCP1493 expression of TACSTD2, EREG, SOX9 and TMEM27 (CLTRN). Color indicates normalized TPM expression; size indicates % of cells expressing a specific gene within a given sample

To investigate the expression patterns of dormancy-associated genes in cancer cells at single-cell resolution, we analyzed the expression of genes positively associated with the *dormant* phenotype: TROP2, SOX9 and EREG and TMEM27 (CLTRN). Single-cell RNA-seq study revealed that all four genes exhibit variable expression levels across samples (Fig. 5A-B). Notably, EREG is widely expressed across samples in a great proportion of cells, while SOX9 seems to be a more effective marker of inter-sample heterogeneity. A different pattern emerges for TMEM2 (CLTRN), which is expressed in most samples, in a small proportion of cells but with variable intensity. Finally, TROP2 exhibits a high discrimination power across samples in a low proportion of cells (consistently < 20%) and a high heterogeneity in intensity. This data indicates that TROP2 expression is confined to a few cancer cells in a subset of patients with variable expression levels, potentially reflecting a resistance-like subpopulation associated with the dormant transcriptional program.

### TROP2 expression in tumor biopsies corroborates the dormant-like phenotype

**Fig. 6:**
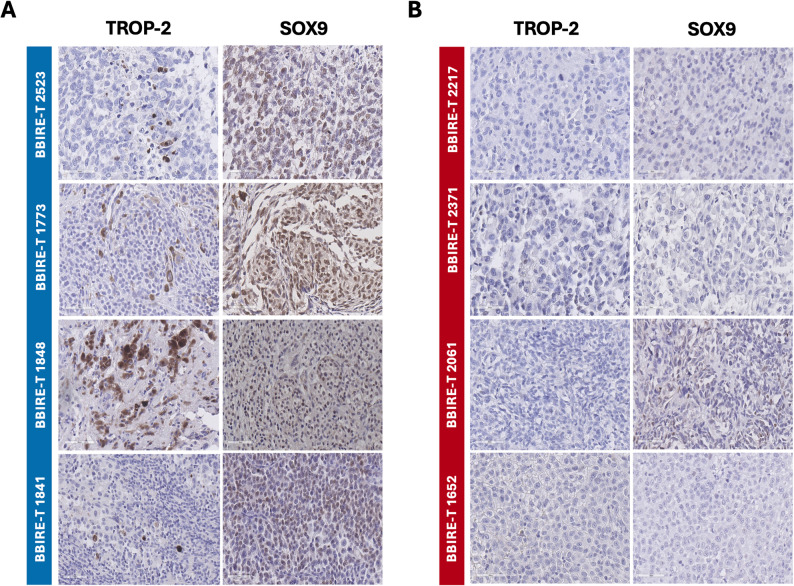
Digital IHC and Immunofluorescence Images of *Dormant* and *Proliferant* Samples. **A** Digital 40× whole-slide scans of four FFPE sections from dormant samples stained with hematoxylin–eosin (H&E), as well as immunostained for the melanoma markers S100, TROP2 and SOX9. **B** Digital 40× whole-slide scans of four FFPE sections from proliferant samples stained with H&E and immunostained for S100, TROP2 and SOX9

Consistent with transcriptomic and single-cell results, immunohistochemical (IHC) analysis performed on our biopsy panel revealed staining for TROP2 in samples classified as dormant-like (Fig. 6A), while proliferative-like samples showed no detectable staining (Fig. 6B). This spatially restricted expression supports the notion that TROP2 is only confined to a limited group of cells within the tumor. Moreover, analysis of the publicly available Protein Atlas resource confirmed that TROP2 protein is expressed in approximately 40–50% of skin cancer samples across three independent cohorts (Figure S5), in agreement with the incidence detected in our dataset (Fig. 3E). Together, these results have validated TROP2 as a possible dormancy-associated marker with restricted but detectable protein-level expression in a clinically relevant subset of melanomas.

### TROP2 localization

TROP2 is classically described as a type I transmembrane glycoprotein involved in cell adhesion, calcium signaling and regulation of proliferation. In epithelial cancers, membranous TROP2 promotes proliferative and pro-tumorigenic signaling through pathways such as ERK/MAPK and PI3K/AKT, contributing to enhanced growth and tumor aggressiveness [36–38]. However, this phenotype is often associated to either the aspecific expression of this gene/protein or the the most comon membrane expression. In contrast, intracellular (cytoplasmic or nuclear) localization of TROP2 appears to reflect alternative functional states. For example, intracellular TROP2 has been associated with poorer responses to immune checkpoint therapy in patients and may reflect internalization or cleavage of the receptor and altered signal transduction [34]. The intracellular domain (TROP2-ICD) released after regulated intramembrane proteolysis can translocate to the nucleus and modulate transcriptional programs linked to proliferation and epithelial–mesenchymal states [39]. Additionally, structural studies show that the intracellular domain (ICD) of TROP2, following proteolytic release, may translocate to the nucleus and modulate cell cycle regulators [40].

**Fig. 7:**
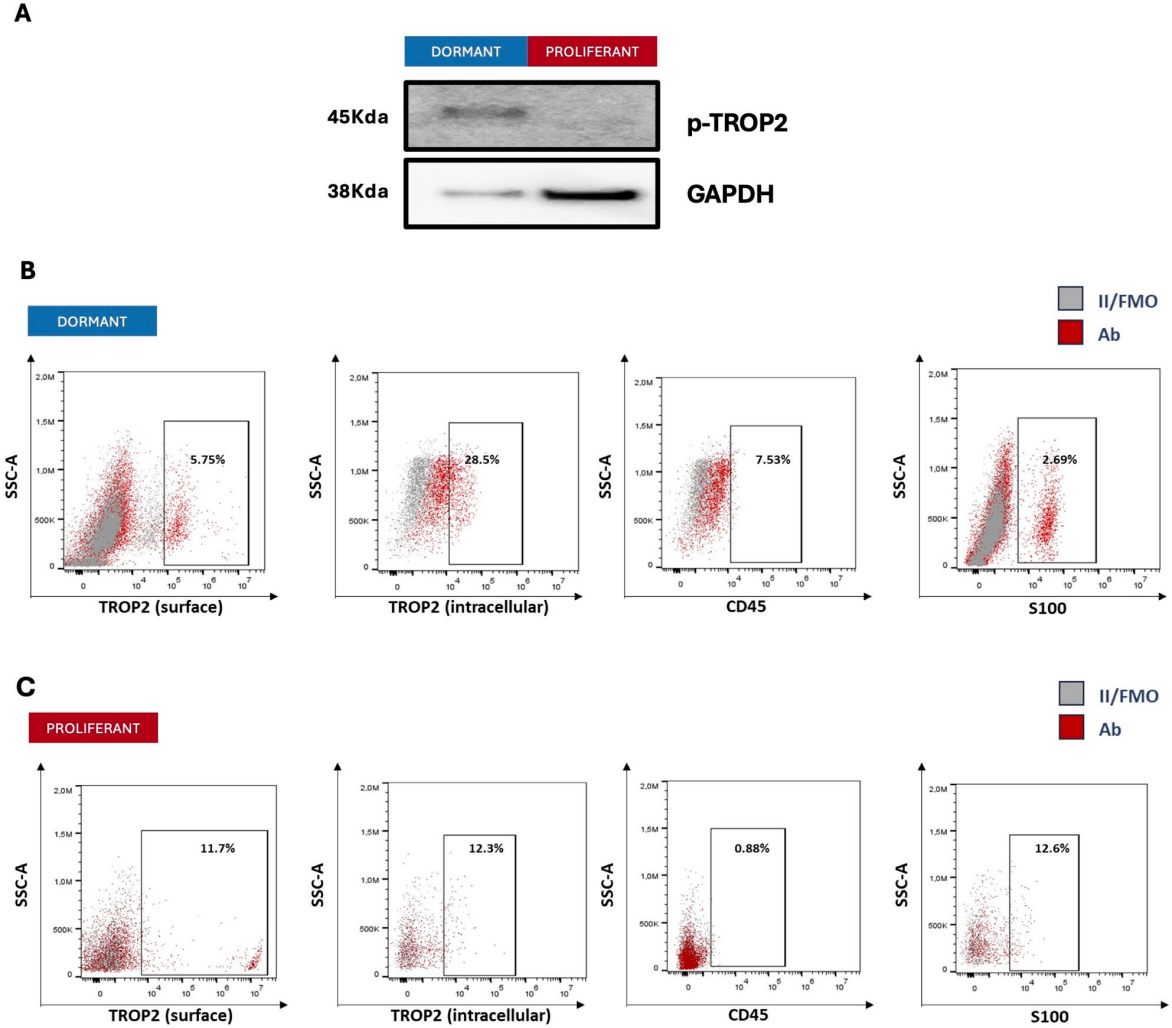
Cellular localization of TROP2. **A** Western blot of 1 dormant and 1 proliferant samples for intracellular and membrane TROP2 (**B**) FACS on intracellular and membrane proportions in a dormant sample (**C**) FACS on intracellular and membrane proportions in a proliferant sample

In our dataset, we evaluated TROP2 localization via flow cytometry and Western blot fractionation in dormant versus proliferative melanoma samples. Western blot analyses of membrane-enriched versus cytoplasmic fractions revealed that dormant cultures exhibited a higher abundance of cytoplasmic TROP2 compared to proliferative ones (Fig. 7A). Similarly, at FACS, beyond an increased overall TROP2 signal in *dormant* cells, we found that *dormant* cells showed a higher proportion of intracellular TROP2 (28.5%) (Fig. 7B) compared with the proliferative group (12.3%) (Fig. 7C) and lower proportion of membranous TROP2 (5.75% vs. 11.7%). These findings suggest that in our model dormant melanoma cells preferentially retain TROP2 in intracellular compartments, which may reflect a distinct signaling state from the *dormant* phenotype.

## Discussion

Despite advances in treatments approaches, metastatic melanoma remains one of the most lethal forms of skin cancer, with a propensity for early dissemination and resistance to therapy [2]. So, the understanding of the molecular mechanisms that drive this adaptive resistance process is critical for improving therapeutic outcomes. EMT is a highly conserved biological process in which epithelial cells lose their polarity and intercellular junctions, acquiring a mesenchymal phenotype with increased motility, invasiveness and resistance to apoptosis. In physiological contexts, EMT is necessary in embryonic development, tissue remodelling and repair [41, 42]. It is also implicated in tumor progression, metastasis and resistance to therapy. Indeed, through this process, epithelial cancer cells acquire mesenchymal features, enhancing their migratory and invasive capabilities while reducing sensitivity to conventional treatments [43]. Although melanoma originates from the neuroectoderm and does not exhibit classical epithelial characteristics, a “pseudo-EMT” phenotype has been described. This state is marked by the downregulation of melanocytic differentiation markers and the acquisition of mesenchymal-like features, mimicking certain aspects of canonical EMT [15, 44]. This phenotypic transition is associated with a dedifferentiated and highly invasive cellular state, which correlates with poor clinical outcomes and increased resistance to therapy [44, 45]. Recent advances in transcriptomic and single-cell technologies have refined the understanding of cell plasticity in melanoma, suggesting that melanoma cells undergo a more nuanced, EMT-like phenotypic modulation that unfolds along a continuous and dynamic spectrum [15, 45–47]. These cells can occupy a range of intermediate phenotypes that co-express transcriptional programs associated with both proliferation and invasion, reflecting a high degree of transcriptional plasticity and functional adaptability. These intermediate EMT-like states actively contribute to therapy resistance and immune evasion. Multiple studies have demonstrated that melanoma cells in these intermediate states are able to escape immune surveillance and exhibit reduced sensitivity to MAPK pathway inhibitors as well as immunotherapy [48–50]. Moreover, in these states, melanoma cells reduce proliferative rate and acquire a drug-tolerant phenotype observed in minimal residual disease following targeted therapy. This therapy-persistent subpopulation is particularly challenging to eradicate and is believed to serve as a reservoir for relapse and acquired resistance [9, 51, 52].

Among the molecular determinants governing phenotypic plasticity in melanoma, a network of transcription factors and surface markers has been identified as principal actors in orchestrating transitions between proliferative and invasive states. Key transcriptional regulators such as SOX9, Zinc finger E-box-binding homeobox 1 (ZEB1) and Microphthalmia-Associated Transcription Factor (MITF) have emerged as central players in this dynamic process [44, 53–55]. MITF is typically associated with a highly proliferative and differentiated state, whereas ZEB1 and SOX9 are preferentially expressed in more invasive and de-differentiated phenotypes [56–58]. The inverse relationship between MITF and these transcription factors reflects a core mechanism by which melanoma cells undergo phenotype switching in response to environmental cues and therapeutic pressures. In addition, several cell surface markers including AXL, nerve growth factor receptor (NGFR) and TROP2 have been shown to delineate distinct cellular subpopulations within melanomas and are increasingly recognized as functional mediators of drug resistance and immune evasion [34, 59, 60]. For instance, high AXL expression is associated with a mesenchymal-like, therapy-resistant state and correlates with poor prognosis in patients receiving MAPK inhibitors [61]. NGFR marks a population with enhanced survival capacity under therapeutic stress [62]. Similarly, TROP2 has been implicated in promoting migration, invasion, and resistance, and may serve as a potential target for antibody–drug conjugates [63]. Importantly, in our study TROP2 exhibited highly coherent RNA and protein expression patterns across our cohort and multiple external datasets, including the *DFCI 2019* cohort, two single-cell datasets and the *Human Protein Atlas*. This consistency supports the robustness of TROP2 biology in melanoma, both in terms of expression intensity and in population incidence (around 50% of melanoma cases). Moreover, two single-cell datasets corroborated our hypothesis about the resistant-cell compartment: although TROP2 is present in roughly 50% of patients, only a very small subset of malignant cells exhibits detectable TROP2 expression, indicating a rare yet potentially functionally relevant subpopulation. These phenotype-associated factors do not act in isolation but are embedded within broader transcriptional and epigenetic programs that interface with inflammatory signalling pathways, metabolic reprogramming and cellular adhesion mechanisms [27].

This study provides evidence for the existence of a phenotypically and transcriptionally distinct dormant state in melanoma, with significant implications for both prognosis and therapeutic responsiveness. Using a systematic and integrative approach that combined matched patient-derived melanoma cell lines with bulk RNA-sequencing of clinical biopsy specimens, we identified a reproducible dichotomy between proliferative and dormant cellular states. The dormant phenotype was consistently characterized by low mitotic activity, reflecting reduced proliferative capacity and by elevated expression of mesenchymal and EMT-associated markers, indicative of a more invasive and drug-tolerant transcriptional program. These features were validated across both in vitro and ex vivo settings, supporting the biological relevance and stability of the dormant state at both the cellular and transcriptional levels. To systematically quantify the dormant phenotype, a composite eight-gene expression signature was developed and used to derive a dormancy score, enabling stratification of independent patient cohorts and publicly available datasets, including the *DFCI 2019* melanoma cohort, along a dormancy–proliferation axis. Application of this transcriptional score revealed a significant association between the dormant phenotype and worse clinical outcomes, including PFS and OS. Furthermore, patients with high dormancy scores exhibited diminished response to immune checkpoint inhibitors, as assessed by RECIST criteria, highlighting the potential of this signature as both a prognostic and predictive biomarker. WGCNA analysis was performed to elucidate the molecular architecture underlying this dormant state. This approach identified a dormancy-associated eigengene module enriched in biological processes antagonistic to proliferation, such as cell cycle arrest and transcriptional repression, yet permissive to migratory and invasive behaviour. Within this module, TROP2 emerged as a central hub gene, suggestive of a regulatory role in maintaining dormancy. TROP2 upregulation was not only prominent at the transcriptomic level but also confirmed at the protein level through digital pathology and immunohistochemistry. Focal TROP2 expression was observed in tumor regions with a dormant-like phenotype, consistent with previous studies implicating TROP2 in tumorigenesis, invasion and drug resistance [64, 65]. Supporting its potential relevance in cutaneous malignancies, TROP2 expression data from The Human Protein Atlas indicated positivity in approximately 40–50% of skin cancers, mirroring the prevalence observed within the study cohort. Additionally, our analysis across bulk and single-cell datasets demonstrates that TROP2 expression is both quantitatively restricted and spatially confined to a rare tumor subpopulation, reinforcing its association with a resistance-linked cellular niche. Further refinement using single-cell RNA sequencing revealed that TROP2 and SOX9 expression were largely restricted to a minor subset of malignant cells, reinforcing the notion of a phenotypically distinct and resistance-associated cellular niche. Beyond expression, the subcellular localization of TROP2 further stratifies this phenotype. While TROP2 is membranous in normal tissues, intracellular accumulation has been described in cancers and associated with altered signalling and stress responses. In our samples, dormant cells exhibited a mixed localization pattern with a predominance of intracellular TROP2 by both IHC and FACS, suggesting that localization may contribute to the functional behaviour of the dormant state and could expand the informative potential of TROP2 as a biomarker. This cellular group likely contributes to long-term tumor persistence by serving as a reservoir of immune-evasive and treatment-refractory clones. These findings are aligned with established models of tumor dormancy, which propose that low-proliferative, yet migratory tumor states mediate escape from immune surveillance and foster therapeutic resistance [66, 67]. Importantly, despite its prognostic significance, the dormancy score did not correlate with CD8⁺ T-cell infiltration, suggesting that the immune escape associated with dormancy may not arise from classical mechanisms of T-cell exclusion or immune suppression. Consistent with this, we observed no specific association between TROP2-expressing tumor regions and immune infiltrate. This is coherent with the fact that TROP2-positive cells represent a very small fraction of the tumor mass, making macroscopic immune effects unlikely. Thus, while a significant association exists between the dormancy signature and reduced response to immunotherapy, this likely reflects an intrinsic resistance program rather than direct modulation of the immune microenvironment.

## Conclusions

This study defines a robust transcriptional program fundamental for melanoma dormancy and identifies a clinically relevant gene signature able to stratify the melanoma patients along a dormancy–proliferation axis. Among the genes identified, TROP2 stands out as a putative biomarker of dormancy and a potential therapeutic target, exhibiting both transcriptomic and protein-level upregulation in dormant-like tumor regions. The dormancy-associated signature demonstrated significant prognostic value, correlating with poor PFS and OS, as well as reduced responsiveness to immune checkpoint blockade. These findings emphasize the clinical relevance of tumor dormancy as a mechanism of therapeutic resistance and immune escape in melanoma. They also underscore the urgent need for prospective clinical validation of dormancy biomarkers and the development of integrative therapeutic strategies aimed at eradicating residual, slow-cycling, immune-evasive tumor cell populations. Targeting the dormant tumor niche represents a promising avenue to overcome minimal residual disease and improve the durability of response in patients receiving conventional and immune-based therapies. In conclusion, if validated, TROP2 could represent a valuable prognostic and predictive biomarker of response to standard therapies in melanoma. Moreover, it may serve as a promising therapeutic target to overcome acquired resistance mechanisms, particularly in tumor subpopulations exhibiting a dormant or drug-tolerant phenotype.

## Supplementary Information

Supplementary Material 1.

## Data Availability

Processed TPM matrix will be published on GEO platform. Code used for the analysis is available at https://github.com/BBIRE/JECCR-Betti-et-al-25. Further information related to the current study is available from the corresponding author upon reasonable request.

## Abbreviations

EMT: Epithelial-to-mesenchymal transition
FFPE: Formalin Fixed Paraffine Embedded
IHC: Immunohistochemistry
MITF: Microphthalmia-Associated Transcription Factor
NGFR: Nerve Growth Factor Receptor
NGS: New Generation Sequencing
OS: Overall Survival
PFS: Progression Free Survival
SOX9: SRY-box transcription factor 9
TPM: Transcript-level expression
VAF: Variant Allele Frequency
WGCNA: Weighted Gene Correlation Network Analysis
ZEB1: Zinc finger E-box-binding homeobox 1

## References

[1] Siegel RL, Giaquinto AN, Jemal A. Cancer statistics, 2024. CA Cancer J Clin. 2024;74(1):12–49. 38230766 10.3322/caac.21820

[2] Falcone I, Conciatori F, Bazzichetto C, Ferretti G, Cognetti F, Ciuffreda L, et al. Tumor microenvironment: implications in melanoma resistance to targeted therapy and immunotherapy. Cancers (Basel). 2020;12(10):2870. 33036192 10.3390/cancers12102870 PMC7601592

[3] Ricciardi E, Giordani E, Ziccheddu G, Falcone I, Giacomini P, Fanciulli M, et al. Metastatic melanoma: liquid biopsy as a new precision medicine approach. Int J Mol Sci. 2023;24(4):4014. 36835424 10.3390/ijms24044014 PMC9962821

[4] Larkin J, Chiarion-Sileni V, Gonzalez R, Grob JJ, Cowey CL, Lao CD, et al. Combined nivolumab and ipilimumab or monotherapy in untreated melanoma. N Engl J Med. 2015;373(1):23–34. 26027431 10.1056/NEJMoa1504030 PMC5698905

[5] Robert C, Schachter J, Long GV, Arance A, Grob JJ, Mortier L, et al. Pembrolizumab versus ipilimumab in advanced melanoma. N Engl J Med. 2015;372(26):2521–32. 25891173 10.1056/NEJMoa1503093

[6] Lazaroff J, Bolotin D. Targeted therapy and immunotherapy in melanoma. Dermatol Clin. 2023;41(1):65–77. 36410984 10.1016/j.det.2022.07.007

[7] Hugo W, Zaretsky JM, Sun L, Song C, Moreno BH, Hu-Lieskovan S, et al. Genomic and transcriptomic features of response to Anti-PD-1 therapy in metastatic melanoma. Cell. 2016;165(1):35–44. 26997480 10.1016/j.cell.2016.02.065 PMC4808437

[8] Shaffer SM, Dunagin MC, Torborg SR, Torre EA, Emert B, Krepler C, et al. Rare cell variability and drug-induced reprogramming as a mode of cancer drug resistance. Nature. 2017;546(7658):431–5. 28607484 10.1038/nature22794 PMC5542814

[9] Rambow F, Rogiers A, Marin-Bejar O, Aibar S, Femel J, Dewaele M, et al. Toward minimal residual Disease-Directed therapy in melanoma. Cell. 2018;174(4):843–55. e19. 30017245 10.1016/j.cell.2018.06.025

[10] Aiello-Couzo NM, Kang Y. A Bridge between melanoma cell States. Nat Cell Biol. 2020;22(8):913–4. 32753670 10.1038/s41556-020-0556-2

[11] Arozarena I, Wellbrock C. Phenotype plasticity as enabler of melanoma progression and therapy resistance. Nat Rev Cancer. 2019;19(7):377–91. 31209265 10.1038/s41568-019-0154-4

[12] Wessely A, Steeb T, Berking C, Heppt MV. How neural crest transcription factors contribute to melanoma Heterogeneity, cellular Plasticity, and treatment resistance. Int J Mol Sci. 2021;22(11):5761. 34071193 10.3390/ijms22115761 PMC8198848

[13] DeGeorgia SN, Kaufman CK. Specific SOX10 enhancer elements modulate phenotype plasticity and drug resistance in melanoma. BioRxiv. 2024.

[14] Tsoi J, Robert L, Paraiso K, Galvan C, Sheu KM, Lay J, et al. Multi-stage differentiation defines melanoma subtypes with differential vulnerability to Drug-Induced Iron-Dependent oxidative stress. Cancer Cell. 2018;33(5):890–904. e5. 29657129 10.1016/j.ccell.2018.03.017 PMC5953834

[15] Pedri D, Karras P, Landeloos E, Marine JC, Rambow F. Epithelial-to-mesenchymal-like transition events in melanoma. FEBS J. 2022;289(5):1352–68. 33999497 10.1111/febs.16021

[16] Bangarh R, Saini RV, Saini AK, Singh T, Joshi H, Ramniwas S, et al. Dynamics of epithelial-mesenchymal plasticity driving cancer drug resistance. Cancer Pathog Ther. 2025;3(2):120–8. 40182126 10.1016/j.cpt.2024.07.002 PMC11963173

[17] Landsberg J, Kohlmeyer J, Renn M, Bald T, Rogava M, Cron M, et al. Melanomas resist T-cell therapy through inflammation-induced reversible dedifferentiation. Nature. 2012;490(7420):412–6. 23051752 10.1038/nature11538

[18] Riesenberg S, Groetchen A, Siddaway R, Bald T, Reinhardt J, Smorra D, et al. MITF and c-Jun antagonism interconnects melanoma dedifferentiation with pro-inflammatory cytokine responsiveness and myeloid cell recruitment. Nat Commun. 2015;6:8755. 26530832 10.1038/ncomms9755 PMC4659938

[19] Muller J, Krijgsman O, Tsoi J, Robert L, Hugo W, Song C, et al. Low MITF/AXL ratio predicts early resistance to multiple targeted drugs in melanoma. Nat Commun. 2014;5:5712. 25502142 10.1038/ncomms6712 PMC4428333

[20] Recasens A, Munoz L. Targeting cancer cell dormancy. Trends Pharmacol Sci. 2019;40(2):128–41. 30612715 10.1016/j.tips.2018.12.004

[21] Ewels PA, Peltzer A, Fillinger S, Patel H, Alneberg J, Wilm A, et al. The nf-core framework for community-curated bioinformatics pipelines. Nat Biotechnol. 2020;38(3):276–8. 32055031 10.1038/s41587-020-0439-x

[22] Love MI, Huber W, Anders S. Moderated Estimation of fold change and dispersion for RNA-seq data with DESeq2. Genome Biol. 2014;15(12):550. 25516281 10.1186/s13059-014-0550-8 PMC4302049

[23] Chen EY, Tan CM, Kou Y, Duan Q, Wang Z, Meirelles GV, et al. Enrichr: interactive and collaborative HTML5 gene list enrichment analysis tool. BMC Bioinformatics. 2013;14:128. 23586463 10.1186/1471-2105-14-128 PMC3637064

[24] Newman AM, Steen CB, Liu CL, Gentles AJ, Chaudhuri AA, Scherer F, et al. Determining cell type abundance and expression from bulk tissues with digital cytometry. Nat Biotechnol. 2019;37(7):773–82. 31061481 10.1038/s41587-019-0114-2 PMC6610714

[25] Chen B, Khodadoust MS, Liu CL, Newman AM, Alizadeh AA. Profiling tumor infiltrating immune cells with CIBERSORT. Methods Mol Biol. 2018;1711:243–59. 29344893 10.1007/978-1-4939-7493-1_12 PMC5895181

[26] Csardi GN. T. The Igraph software package for complex network Research. InterJournal 2006. Complex Syst, 1695. 2006.

[27] Jerby-Arnon L, Shah P, Cuoco MS, Rodman C, Su MJ, Melms JC, et al. A cancer cell program promotes T cell exclusion and resistance to checkpoint Blockade. Cell. 2018;175(4):984–97. e24. 30388455 10.1016/j.cell.2018.09.006 PMC6410377

[28] Prakadan SM, Alvarez-Breckenridge CA, Markson SC, Kim AE, Klein RH, Nayyar N, et al. Genomic and transcriptomic correlates of immunotherapy response within the tumor microenvironment of leptomeningeal metastases. Nat Commun. 2021;12(1):5955. 34642316 10.1038/s41467-021-25860-5 PMC8511044

[29] Uhlen M, Fagerberg L, Hallstrom BM, Lindskog C, Oksvold P, Mardinoglu A, et al. Proteomics. Tissue-based map of the human proteome. Science. 2015;347(6220):1260419. 25613900 10.1126/science.1260419

[30] Simiczyjew A, Kot M, Majkowski M, Zietek M, Matkowski R, Nowak D. Phenotype switching in highly invasive resistant to Vemurafenib and Cobimetinib melanoma cells. Cell Commun Signal. 2025;23(1):449. 41121376 10.1186/s12964-025-02452-0 PMC12542628

[31] Sperber L, von Brandenstein M, Kessler C, Heidenreich J, Storz E, Pfister D, et al. Expression and therapeutic potential of TROP2 in cisplatin-resistant germ cell tumors. J Cancer Res Clin Oncol. 2025;151(11):279. 41055771 10.1007/s00432-025-06325-4 PMC12504156

[32] Aslemarz A, Fagotto-Kaufmann M, Ruppel A, Fagotto-Kaufmann C, Balland M, Lasko P, et al. An EpCAM/Trop2 mechanostat differentially regulates collective behaviour of human carcinoma cells. EMBO J. 2025;44(1):75–106. 39572744 10.1038/s44318-024-00309-9 PMC11696905

[33] Sun Y, Yinwang E, Wang S, Wang Z, Wang F, Xue Y, et al. Phenotypic and Spatial heterogeneity of CD8(+) tumour infiltrating lymphocytes. Mol Cancer. 2024;23(1):193. 39251981 10.1186/s12943-024-02104-w PMC11382426

[34] Bessede A, Peyraud F, Besse B, Cousin S, Cabart M, Chomy F, et al. TROP2 is associated with primary resistance to immune checkpoint Inhibition in patients with advanced Non-Small cell lung cancer. Clin Cancer Res. 2024;30(4):779–85. 38048058 10.1158/1078-0432.CCR-23-2566 PMC10870116

[35] Sl NJJT, Gt N. Tertiary lymphoid structures and B lymphocytes in cancer prognosis and response to immunotherapies. Oncoimmunology. 2021;10(1):1900508. 33854820 10.1080/2162402X.2021.1900508 PMC8018489

[36] Trerotola M, Jernigan DL, Liu Q, Siddiqui J, Fatatis A, Languino LR. Trop-2 promotes prostate cancer metastasis by modulating beta(1) integrin functions. Cancer Res. 2013;73(10):3155–67. 23536555 10.1158/0008-5472.CAN-12-3266 PMC3655712

[37] Cubas R, Zhang S, Li M, Chen C, Yao Q. Trop2 expression contributes to tumor pathogenesis by activating the ERK MAPK pathway. Mol Cancer. 2010;9:253. 20858281 10.1186/1476-4598-9-253 PMC2946292

[38] Goldenberg DM, Stein R, Sharkey RM. The emergence of trophoblast cell-surface antigen 2 (TROP-2) as a novel cancer target. Oncotarget. 2018;9(48):28989–9006. 29989029 10.18632/oncotarget.25615 PMC6034748

[39] Trerotola M, Cantanelli P, Guerra E, Tripaldi R, Aloisi AL, Bonasera V, et al. Upregulation of Trop-2 quantitatively stimulates human cancer growth. Oncogene. 2013;32(2):222–33. 22349828 10.1038/onc.2012.36

[40] Liu X, Li J, Deng J, Zhao J, Zhao G, Zhang T, et al. Targeting Trop2 in solid tumors: a look into structures and novel epitopes. Front Immunol. 2023;14:1332489. 38179054 10.3389/fimmu.2023.1332489 PMC10765514

[41] Thiery JP, Sleeman JP. Complex networks orchestrate epithelial-mesenchymal transitions. Nat Rev Mol Cell Biol. 2006;7(2):131–42. 16493418 10.1038/nrm1835

[42] Lamouille S, Xu J, Derynck R. Molecular mechanisms of epithelial-mesenchymal transition. Nat Rev Mol Cell Biol. 2014;15(3):178–96. 24556840 10.1038/nrm3758 PMC4240281

[43] Gundamaraju R, Lu W, Paul MK, Jha NK, Gupta PK, Ojha S, et al. Autophagy and EMT in cancer and metastasis: who controls whom? Biochim Biophys Acta Mol Basis Dis. 2022;1868(9):166431. 35533903 10.1016/j.bbadis.2022.166431

[44] Hossain SM, Eccles MR. Phenotype switching and the melanoma Microenvironment; impact on immunotherapy and drug resistance. Int J Mol Sci. 2023;24(2):1601. 36675114 10.3390/ijms24021601 PMC9864717

[45] Bhat GR, Sethi I, Sadida HQ, Rah B, Mir R, Algehainy N, et al. Cancer cell plasticity: from cellular, molecular, and genetic mechanisms to tumor heterogeneity and drug resistance. Cancer Metastasis Rev. 2024;43(1):197–228. 38329598 10.1007/s10555-024-10172-z PMC11016008

[46] Rambow F, Marine JC, Goding CR. Melanoma plasticity and phenotypic diversity: therapeutic barriers and opportunities. Genes Dev. 2019;33(19–20):1295–318. 31575676 10.1101/gad.329771.119 PMC6771388

[47] Brabletz S, Schuhwerk H, Brabletz T, Stemmler MP. Dynamic EMT: a multi-tool for tumor progression. EMBO J. 2021;40(18):e108647. 34459003 10.15252/embj.2021108647 PMC8441439

[48] Tang Y, Durand S, Dalle S, Caramel J. EMT-Inducing transcription Factors, drivers of melanoma phenotype Switching, and resistance to treatment. Cancers (Basel). 2020;12(8):2154. 32759677 10.3390/cancers12082154 PMC7465730

[49] Zielinska MK, Ciazynska M, Sulejczak D, Rutkowski P, Czarnecka AM. Mechanisms of resistance to Anti-PD-1 immunotherapy in melanoma and strategies to overcome it. Biomolecules. 2025;15(2):269. 40001572 10.3390/biom15020269 PMC11853485

[50] Benboubker V, Boivin F, Dalle S, Caramel J. Cancer cell phenotype plasticity as a driver of immune escape in melanoma. Front Immunol. 2022;13:873116. 35432344 10.3389/fimmu.2022.873116 PMC9012258

[51] Marin-Bejar O, Rogiers A, Dewaele M, Femel J, Karras P, Pozniak J, et al. Evolutionary predictability of genetic versus nongenetic resistance to anticancer drugs in melanoma. Cancer Cell. 2021;39(8):1135–e498. 34143978 10.1016/j.ccell.2021.05.015

[52] Patel RP, Somasundram PM, Smith LK, Sheppard KE, McArthur GA. The therapeutic potential of targeting minimal residual disease in melanoma. Clin Transl Med. 2023;13(3):e1197. 36967556 10.1002/ctm2.1197 PMC10040726

[53] Debnath P, Huirem RS, Dutta P, Palchaudhuri S. Epithelial-mesenchymal transition and its transcription factors. Biosci Rep. 2022;42(1):BSR20211754. 34708244 10.1042/BSR20211754 PMC8703024

[54] Carrasco-Garcia E, Lopez L, Moncho-Amor V, Carazo F, Aldaz P, Collado M, et al. SOX9 triggers different epithelial to mesenchymal transition States to promote pancreatic cancer progression. Cancers (Basel). 2022;14(4):916. 35205666 10.3390/cancers14040916 PMC8870732

[55] Huang JQ, Wei FK, Xu XL, Ye SX, Song JW, Ding PK, et al. SOX9 drives the epithelial-mesenchymal transition in non-small-cell lung cancer through the Wnt/beta-catenin pathway. J Transl Med. 2019;17(1):143. 31060551 10.1186/s12967-019-1895-2 PMC6501400

[56] Hartman ML, Czyz M. MITF in melanoma: mechanisms behind its expression and activity. Cell Mol Life Sci. 2015;72(7):1249–60. 25433395 10.1007/s00018-014-1791-0 PMC4363485

[57] Durand S, Tang Y, Pommier RM, Benboubker V, Grimont M, Boivin F, et al. ZEB1 controls a lineage-specific transcriptional program essential for melanoma cell state transitions. Oncogene. 2024;43(20):1489–505. 38519642 10.1038/s41388-024-03010-7 PMC11090790

[58] Subhadarshini S, Sahoo S, Debnath S, Somarelli JA, Jolly MK. Dynamical modeling of proliferative-invasive plasticity and IFNgamma signaling in melanoma reveals mechanisms of PD-L1 expression heterogeneity. J Immunother Cancer. 2023;11(9):e0006766. 10.1136/jitc-2023-006766 PMC10496669 37678920

[59] Kharouf N, Flanagan TW, Hassan SY, Shalaby H, Khabaz M, Hassan SL, et al. Tumor microenvironment as a therapeutic target in melanoma treatment. Cancers (Basel). 2023;15(12):3147. 37370757 10.3390/cancers15123147 PMC10296288

[60] Somasundaram R, Villanueva J, Herlyn M. Intratumoral heterogeneity as a therapy resistance mechanism: role of melanoma subpopulations. Adv Pharmacol. 2012;65:335–59. 22959031 10.1016/B978-0-12-397927-8.00011-7 PMC3677516

[61] Nyakas M, Fleten KG, Haugen MH, Engedal N, Sveen C, Farstad IN, et al. AXL Inhibition improves BRAF-targeted treatment in melanoma. Sci Rep. 2022;12(1):5076. 35332208 10.1038/s41598-022-09078-z PMC8948193

[62] Huang F, Santinon F, Flores Gonzalez RE, Del Rincon SV. Melanoma plasticity: promoter of metastasis and resistance to therapy. Front Oncol. 2021;11:756001. 34604096 10.3389/fonc.2021.756001 PMC8481945

[63] Wang J, Zhang Y, Bai R, Wu Y, Tong Z, Liu A, et al. Novel TROP2 antibody-drug conjugates for treatment of HER2-negative metastatic breast cancer patients with brain metastases: a promising option(☆). ESMO Open. 2025;10(5):105059. 40359710 10.1016/j.esmoop.2025.105059 PMC12141909

[64] Shvartsur A, Bonavida B. Trop2 and its overexpression in cancers: regulation and clinical/therapeutic implications. Genes Cancer. 2015;6(3–4):84–105. 26000093 10.18632/genesandcancer.40 PMC4426947

[65] Koltai T, Fliegel L. The relationship between Trop-2, chemotherapeutic Drugs, and chemoresistance. Int J Mol Sci. 2023;25(1):87. 38203255 10.3390/ijms25010087 PMC10779383

[66] Gomis RR, Gawrzak S. Tumor cell dormancy. Mol Oncol. 2017;11(1):62–78. 28017284 10.1016/j.molonc.2016.09.009 PMC5423221

[67] Goddard ET, Linde MH, Srivastava S, Klug G, Shabaneh TB, Iannone S, et al. Immune evasion of dormant disseminated tumor cells is due to their scarcity and can be overcome by T cell immunotherapies. Cancer Cell. 2024;42(1):119–34. e12. 38194912 10.1016/j.ccell.2023.12.011 PMC10864018

