# Supplementary material for: Biopsy RNA-seq captures TROP-2–linked migration and clonal resistance to forecast aggressiveness in metastatic melanoma
Source: J Exp Clin Cancer Res. 2026 Jan 28;45:59. doi: 10.1186/s13046-026-03646-1 (PMC12934100; doi:10.1186/s13046-026-03646-1)
Supplement: Supplementary file 1 — Supplementary Material 1. [file 13046_2026_3646_MOESM1_ESM.docx]

**Supplementary Figures**

**S1.** Exemplary phenotypic characterization of BRAF **(A)** and NRAS **(B)** in tissues vs. passage 2 cell lines in *dormant* samples (blue) and *proliferant* samples (red)

**S2.** **(A)** Cell morphology in a dormant cell line **(B)** Digital scan of dormant sample FFPE tissue at 40× resolution, stained with H&E and with the proliferation marker Ki67 **(C)** Cell morphology in a proliferant cell line **(D)** Digital scan of proliferant sample FFPE tissue at 40× resolution, stained with H&E and with the proliferation marker Ki67.

**S3.** Enriched pathways in dormancy associated gene module


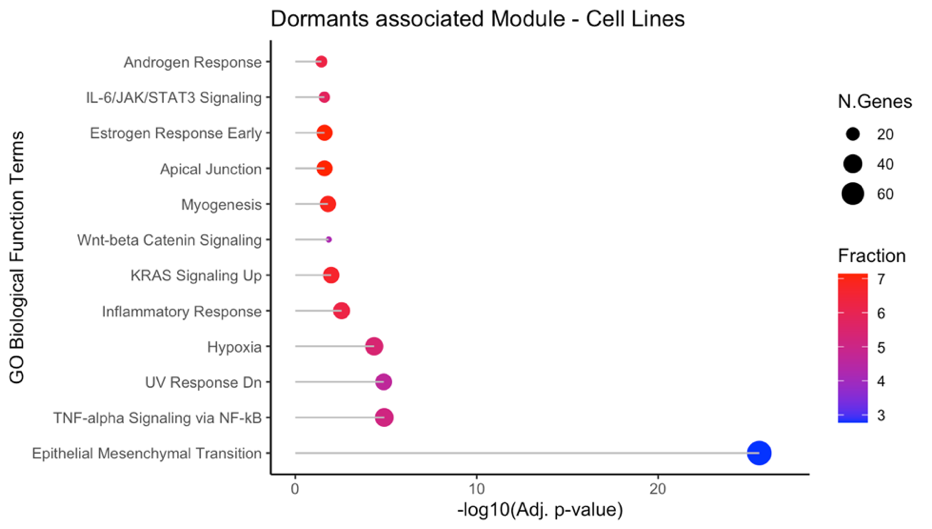


**S4.** **(A)** Recist derived description of response to Immunotherapy for the *dfci 2019* cohort in *dormants* vs. *proliferants* **(B)** Relative immune abundances estimated via CIBERSORTx, in *dormants* vs. *proliferants* **(C)** IHC performed on digital pathological slides in 2 exemplary *dormant* (blue) and *proliferant* (red) samples for CD20 (B cell marker) and CD8 (T cell cCD8+ marker).

**S5.** Protein Atlas TROP2 protein expression incidence across cancer types in three cohorts.

**A**
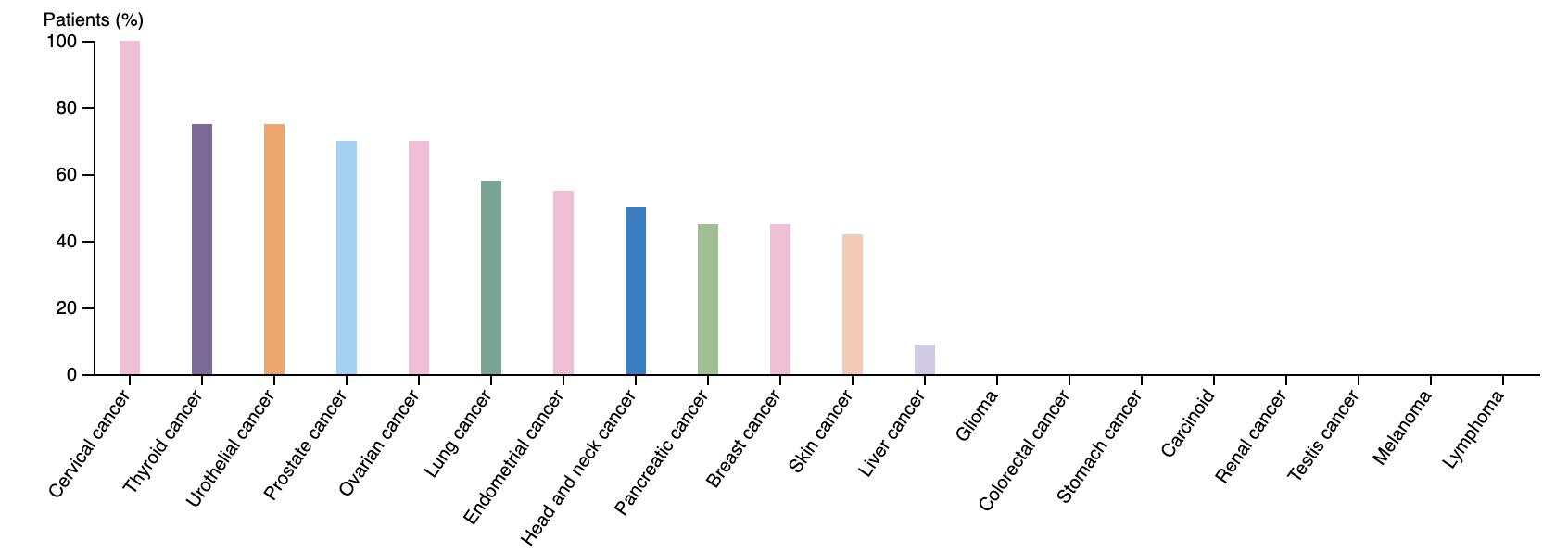
**B**
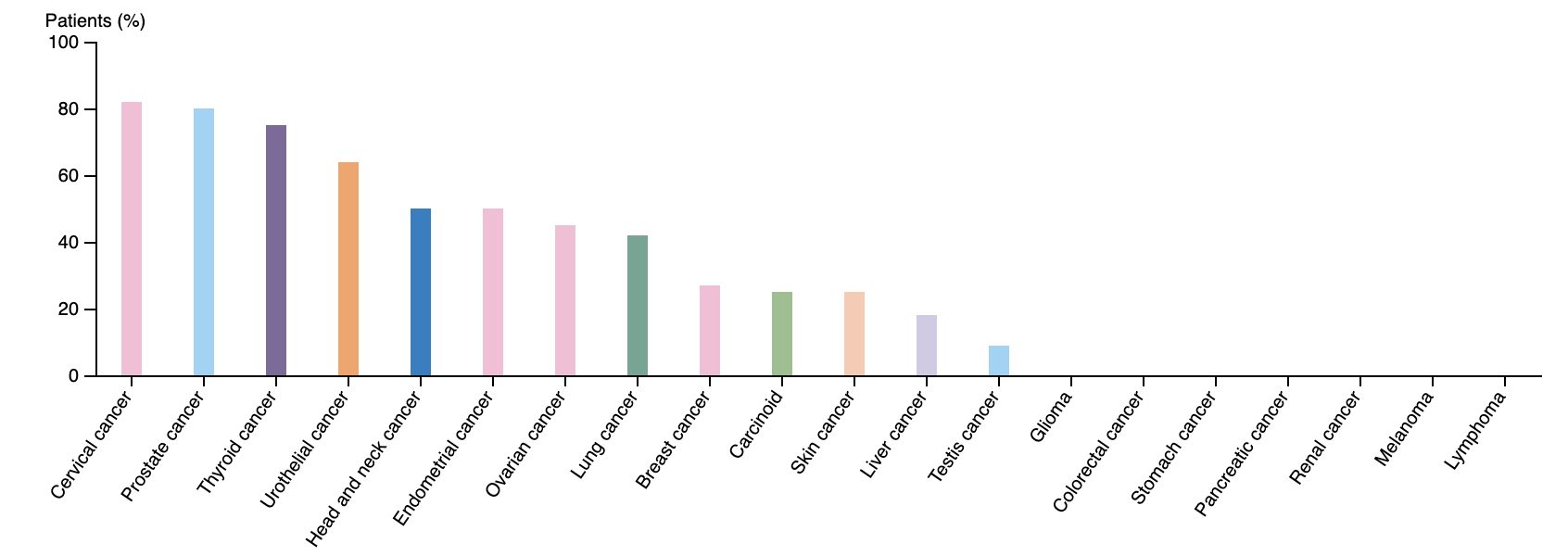
 **C**
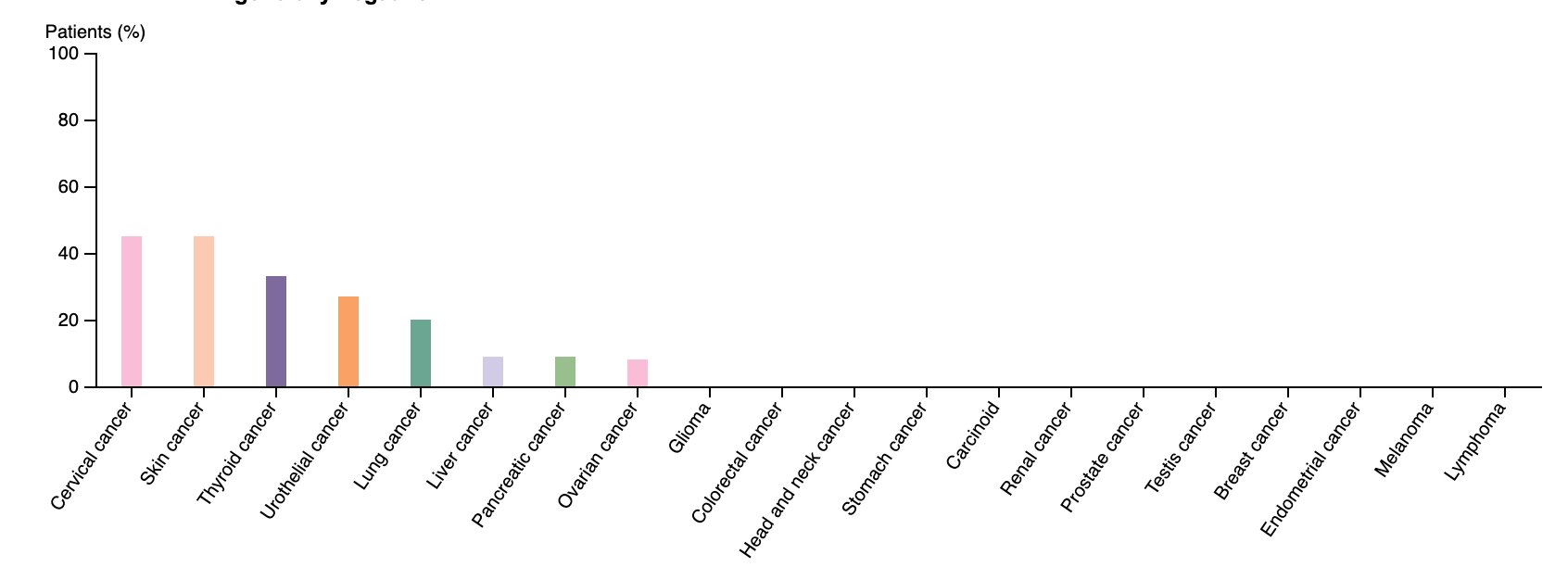


**Supplementary Tables**

**Table S1.** DE gene list.

| **GENE** | **baseMean** | **log2FC** | **lfcSE** | **stat** | **pvalue** | **padj** |
| --- | --- | --- | --- | --- | --- | --- |
| **ASB4** | 167,1197333 | 4,77505449 | 1,264510023 | 3,776209287 | 0,000159233 | 0,046471152 |
| **DEF6** | 205,2525086 | 1,070459629 | 0,264338151 | 4,049584309 | 5,13E-05 | 0,024182719 |
| **PRSS8** | 9,23290982 | -4,011446435 | 0,961979474 | -4,169991714 | 3,05E-05 | 0,020425679 |
| **SNCB** | 17,82470104 | 3,338374166 | 0,803899781 | 4,152724312 | 3,29E-05 | 0,020425679 |
| **DHRS2** | 43,9259176 | 3,440790798 | 0,915244531 | 3,759422409 | 0,000170306 | 0,046471152 |
| **COL20A1** | 60,07794247 | 4,107383355 | 0,977016835 | 4,204004689 | 2,62E-05 | 0,020141475 |
| **LAMA1** | 1317,7434 | 2,924071772 | 0,652674514 | 4,480137818 | 7,46E-06 | 0,01104963 |
| **BEX4** | 329,7568921 | -1,419099994 | 0,37628283 | -3,771365265 | 0,000162357 | 0,046471152 |
| **C7orf63** | 82,35344497 | -1,009435761 | 0,256320318 | -3,938180823 | 8,21E-05 | 0,032125007 |
| **KRT23** | 20,28779782 | -4,663656793 | 1,170097422 | -3,985699571 | 6,73E-05 | 0,02847522 |
| **LRP2BP** | 55,12703967 | -1,960196841 | 0,485045709 | -4,041262101 | 5,32E-05 | 0,024500471 |
| **LPPR4** | 204,3230667 | -3,155791183 | 0,682407057 | -4,624499628 | 3,76E-06 | 0,007954768 |
| **EREG** | 73,20892766 | -4,447895767 | 0,970708433 | -4,582113037 | 4,60E-06 | 0,007954768 |
| **SOX9** | 415,3421039 | -2,682862285 | 0,681153495 | -3,938704426 | 8,19E-05 | 0,032125007 |
| **FAM78A** | 501,6236804 | 1,138680742 | 0,271532276 | 4,193537347 | 2,75E-05 | 0,020340855 |
| **CPA4** | 23,03013149 | -3,377749663 | 0,773339987 | -4,367742159 | 1,26E-05 | 0,012397134 |
| **VGF** | 935,064248 | 4,152018559 | 0,804933236 | 5,158214833 | 2,49E-07 | 0,001292576 |
| **FIBCD1** | 9,938791739 | 3,685375803 | 0,856085721 | 4,304914466 | 1,67E-05 | 0,014434535 |
| **FAM83F** | 29,43788961 | -3,839978824 | 0,945262281 | -4,062342168 | 4,86E-05 | 0,024083316 |
| **EHF** | 31,70524719 | -2,311645203 | 0,618980957 | -3,734598259 | 0,000188015 | 0,049987906 |
| **CLCA2** | 26,59333038 | -4,548791015 | 0,987312161 | -4,607247026 | 4,08E-06 | 0,007954768 |
| **AMIGO2** | 159,9038271 | 2,015560213 | 0,44845554 | 4,494448242 | 6,98E-06 | 0,01104963 |
| **NKD1** | 230,3911446 | -2,445678071 | 0,556817917 | -4,392240259 | 1,12E-05 | 0,012397134 |
| **PMAIP1** | 238,1502009 | -2,075976493 | 0,524539448 | -3,957712808 | 7,57E-05 | 0,031385247 |
| **FLG** | 81,37579764 | -4,699774767 | 1,202382342 | -3,908719053 | 9,28E-05 | 0,033991458 |
| **ANKRD53** | 14,10687796 | -1,856405271 | 0,457702172 | -4,055924105 | 4,99E-05 | 0,024083316 |
| **GRIP2** | 87,10657382 | 2,463472618 | 0,62924322 | 3,914976816 | 9,04E-05 | 0,033991458 |
| **TMEM27** | 9,382445183 | -2,762447926 | 0,660381025 | -4,183112207 | 2,88E-05 | 0,020425679 |
| **MAL2** | 33,35366023 | -3,69674463 | 0,947191446 | -3,902848412 | 9,51E-05 | 0,033991458 |
| **MPZL2** | 151,0839499 | -2,203435546 | 0,529911457 | -4,158120223 | 3,21E-05 | 0,020425679 |
| **MPV17L** | 86,49732826 | -2,123335952 | 0,516127717 | -4,113973889 | 3,89E-05 | 0,021224003 |
| **SHANK2** | 30,27905793 | -3,200732595 | 0,739471906 | -4,328403244 | 1,50E-05 | 0,013683077 |
| **SERPINB7** | 16,39716332 | -5,840104746 | 1,334892136 | -4,374963779 | 1,21E-05 | 0,012397134 |
| **MYO5B** | 304,9546553 | 2,509552567 | 0,52099167 | 4,816876563 | 1,46E-06 | 0,006048148 |
| **KRT1** | 63,06781102 | -5,921529345 | 1,500009986 | -3,947659949 | 7,89E-05 | 0,032090547 |
| **MGAT5B** | 267,8962411 | 3,040930347 | 0,73422131 | 4,141708103 | 3,45E-05 | 0,020425679 |
| **LRP1B** | 49,3191563 | -4,009061133 | 1,043001498 | -3,84377313 | 0,000121157 | 0,041189414 |
| **SLC9C1** | 26,63818945 | 3,356432174 | 0,877941229 | 3,823071595 | 0,000131799 | 0,043385045 |
| **NIPAL4** | 26,44152823 | -3,528247264 | 0,767743915 | -4,595604336 | 4,31E-06 | 0,007954768 |
| **SFN** | 45,68046727 | -5,085239743 | 1,328134615 | -3,828858675 | 0,000128739 | 0,043061087 |
| **MUC20** | 91,53145051 | 1,626838789 | 0,398896796 | 4,078345091 | 4,54E-05 | 0,023735252 |
| **SPNS3** | 9,791445855 | 3,06932787 | 0,698508783 | 4,394114929 | 1,11E-05 | 0,012397134 |
| **TACSTD2** | 37,47853446 | -3,226842735 | 0,777148164 | -4,15215899 | 3,29E-05 | 0,020425679 |
| **SPATC1** | 6,464052631 | 1,992885017 | 0,525425662 | 3,792896241 | 0,0001489 | 0,046471152 |
| **CLDN4** | 14,61607895 | -2,831642841 | 0,747530793 | -3,787994913 | 0,000151868 | 0,046471152 |
| **MME** | 579,2573369 | 2,763806756 | 0,732253939 | 3,774382912 | 0,000160404 | 0,046471152 |
| **ADAM32** | 74,57642772 | -1,867261517 | 0,493734263 | -3,781916016 | 0,000155626 | 0,046471152 |
| **RYR2** | 191,4878699 | -2,193185001 | 0,532315276 | -4,120086532 | 3,79E-05 | 0,021224003 |
| **L1CAM** | 768,6109587 | 2,692413031 | 0,69558546 | 3,870714939 | 0,000108517 | 0,038142675 |
| **FAM106A** | 100,3789515 | -1,856248147 | 0,491613794 | -3,775826005 | 0,000159478 | 0,046471152 |
| **LCNL1** | 106,9491594 | 2,479495084 | 0,602652687 | 4,114301882 | 3,88E-05 | 0,021224003 |
| **KNOP1P4** | 5,152631696 | 4,328815518 | 1,108533162 | 3,904994155 | 9,42E-05 | 0,033991458 |
| **GHRLOS** | 54,23109618 | 1,414425182 | 0,346997548 | 4,076182063 | 4,58E-05 | 0,023735252 |
| **KRT18P31** | 33,85622903 | 1,2878118 | 0,34239054 | 3,761236513 | 0,000169075 | 0,046471152 |
